# Microglia depletion diminishes key elements of the leukotriene pathway in the brain of Alzheimer’s Disease mice

**DOI:** 10.1186/s40478-020-00989-4

**Published:** 2020-08-08

**Authors:** J. Michael, M. S. Unger, R. Poupardin, P. Schernthaner, H. Mrowetz, J. Attems, L. Aigner

**Affiliations:** 1 Institute of Molecular Regenerative Medicine, Paracelsus Medical University, Strubergasse 21, 5020 Salzburg, Austria; 2 Spinal Cord Injury and Tissue Regeneration Center Salzburg (SCI-TReCS), Paracelsus Medical University, Salzburg, Austria; 3 Experimental and Clinical Cell Therapy Institute, Paracelsus Medical University, Salzburg, Austria; 4 Institute of Neuroscience, Newcastle University, Newcastle upon Tyne, UK; 5 Austrian Cluster for Tissue Regeneration, Vienna, Austria

**Keywords:** Leukotrienes, Cysteinyl-leukotrienes, Alzheimer’s disease, Microglia, RNAseq

## Abstract

Leukotrienes (LTs) contribute to the neuropathology of chronic neurodegenerative disorders including Alzheimer’s Disease (AD), where they mediate neuroinflammation and neuronal cell-death. In consequence, blocking the action of Leukotrienes (LTs) ameliorates pathologies and improves cognitive function in animal models of neurodegeneration. Surprisingly, the source of Leukotrienes (LTs) in the brain is largely unknown. Here, we identified the Leukotriene (LT) synthesis rate-limiting enzyme 5-Lipoxygenase (5-Lox) primarily in neurons and to a lesser extent in a subpopulation of microglia in human Alzheimer´s Disease (AD) hippocampus brain sections and in brains of APP Swedish PS1 dE9 (APP-PS1) mice, a transgenic model for Alzheimer´s Disease (AD) pathology. The 5-Lipoxygenase (5-Lox) activating protein (FLAP), which anchors 5-Lipoxygenase (5-Lox) to the membrane and mediates the contact to the substrate arachidonic acid, was confined exclusively to microglia with the entire microglia population expressing 5-Lipoxygenase activating protein (FLAP). To define the contribution of microglia in the Leukotriene (LT) biosynthesis pathway, we ablated microglia using the colony stimulating factor 1 receptor (CSF1R) inhibitor PLX5622 in wildtype (WT) and APP-PS1 mice. Microglia ablation not only diminished the expression of FLAP and of the Leukotriene (LT) receptor Cysteinylleukotriene receptor 1 (CysLTR1), as expected based on their microglia cell type-specific expression, but also drastically reduced 5-Lipoxygenase (5-Lox) mRNA expression in the brain and its protein expression in neurons, in particular in wildtype (WT) mice. In conclusion i) microglia are key in Leukotriene (LT) biosynthesis, and ii) they regulate neuronal 5-Lipoxygenase (5-Lox) expression implying a yet unknown signaling mechanism between neurons and microglia.

## Introduction

Leukotrienes (LTs) are elevated in the brain in aging, after injury, and in neurodegenerative diseases such as Alzheimer’s Disease (AD) [1–4]. Increased levels of LTs contribute to age- and disease-related brain pathologies such as i) neuroinflammation and microglia / astroglia activation [5–7], ii) neuronal damage [8, 9], and iii) blood-brain-barrier (BBB) permeability [10–12]. Therefore, LT signaling has been recognized as therapeutic target in acute and chronic neurodegenerative diseases (for review see [13, 14]). In the context of AD, various in vivo studies demonstrated that genetic and pharmacological interventions reducing LT production or LT signaling ameliorate pathological burdens such as amyloid and tau load and improve cognitive function [2, 15–18]. Moreover, human genetics data illustrated that a single nucleotide polymorphism in the gene for 5-lox activating protein (FLAP), a key protein in the activation of LT synthesis, correlates with an increased risk for AD suggesting that LTs might indeed contribute to AD development [19]. Vice versa, a Norwegian population-based study suggested that Montelukast, an approved anti-asthmatic drug and LT receptor antagonist, might lower the risk for developing dementias [20]. Finally, a recent case study with 17 dementia patients illustrated beneficial effects of Montelukast on cognition and agitation [21] suggesting that blockade of LT synthesis or LT signaling might indeed be a therapeutic approach for the treatment of dementias such as AD (reviewed in [13]).

**Fig. 1:**
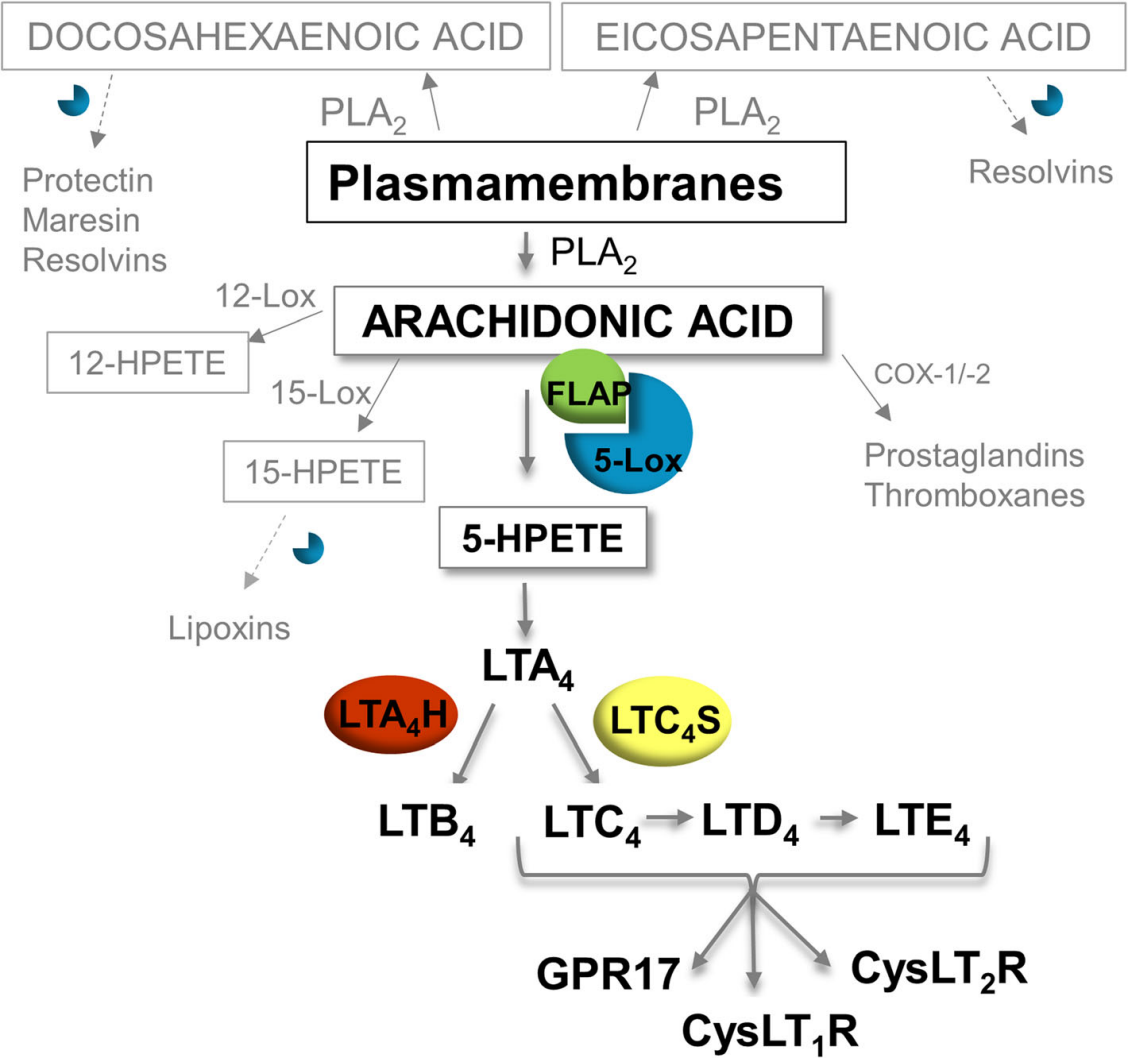
LT biosynthesis is part of metabolism of the omega 6 polyunsaturated arachidonic acid (AA), which is cleaved from plasmamembranes by phospholipase A_2_ (PLA_2_). AA is a substrate for several lipoxygenases. For the production of LTs it is first converted into 5-hydroxy-peroxy-eicosatetraenoic acid (5-HPETE) and afterwards into LTA4, both steps mediated by a complex of the enzyme 5-lipoxygenase (5-Lox) and 5-lipoxygenase activating protein (FLAP) [22]. From LTA4 arise either Leukotriene B4 (LTB4) formed by epoxide hydrolase or Leukotriene C4 (LTC4) formed by LTC4 synthase, which can be further metabolized to LTD4 and LTE4 [23]. LTC4, LTD4 and LTE4 are summarized under the term cysteinyl-leukotrienes (CysLTs). CysLTs display different affinities towards the receptors cysteinyl-leukotriene receptor 1 and 2 (CysLT1R [24]), CysLT2R [25]) and G-protein coupled receptor 17 (GPR17 [26, 27]). The enzyme 5-Lox is also involved in some steps of biosynthesis of specialized pro-resolving mediators from omega 3 poly-unsaturated fatty acids (DHA – docosahexaenoic acid; EPA – eicosapentaenoic acid)

LT biosynthesis is part of the complex metabolism of poly-unsaturated fatty acids (PUFAs), which also includes the production of anti-inflammatory substances (lipoxins, resolvins, maresin and protectin), as well as pro-inflammatory substances like prostaglandins and thromboxanes (Fig. 1). LTs originate from omega-6 poly-unsaturated arachidonic acid (AA), which is converted to LTA4 mediated by the enzyme 5-lipoxygenase (5-Lox) and the activating protein FLAP. These key elements need to be in physical proximity and form a complex to initiate the first reaction of the LT biosynthesis pathway [22]. From LTA4 either LTB4 or cysteinyl-LTs (CysLTs) are generated. CysLTs bind with different affinities to the cysteinyl LT receptor 1 and 2 (CysLT1R [24], CysLT2R [25]) and G-protein coupled receptor 17 (GPR17) [26]. 5-Lox, together with other lipoxygenases (12-Lox and 15-Lox) also plays a role in other pathways of PUFA metabolism, for example in the formation of resolvins starting from the omega-3 poly-unsaturated docosahexaenoic (DHA) or eicosapentaenoic (EPA) acids, which, like AA are also cleaved from plasma membranes by the enzyme Phospholipase A_2_ (PLA_2_) (Fig. 1).

In the periphery LTs are generated by leukocytes [28] with subgroups having different capacities to produce either LTB4 (neutrophils) or CysLTs (eosinophils, basophils) or both (macrophages, mast cells) [29]. In the brain, although there is an age- and disease related increase in LTs, and effects of LTs via CyslTR1 and 2 on various brain cells are well documented (reviewed in [14]), the cellular source of LTs is rather unexplored. Obviously, such knowledge might improve the further understanding of the underlying molecular and cellular pathomechanisms, moreover, it might foster therapy development for diseases such as AD. Prominent neuronal expression of 5-Lox and of FLAP mRNA was described in the hippocampus and in various other regions of the rat brain using in-situ hybridization [30]. Here, we investigated expression of 5-Lox, the rate limiting enzyme in LT production, and of FLAP, the 5-Lox activating protein, in the brain of wild-type (WT) and of transgenic AD mice (APP-PS1 mice) as well as in human post-mortem brain specimen of AD patients. We identified microglia as the only cell type in the brain to express FLAP, while 5-Lox was predominantly expressed in neurons with some expression also in a population of microglia. As an experimental approach, we ablated microglia cells for 4 weeks from the brain of WT and APP-PS1 transgenic mice and analyzed the molecular consequences in terms of the LT pathway.

## Material and methods

### Compounds

PLX5622 was provided by Plexxikon Inc. and formulated in AIN-76A standard chow by Research Diets Inc. at 1200 mg/kg, as previously described [31, 32].

### Animals

Female and male APP Swedish PS1 dE9 mice (reviewed in [33] and [34]) expressing a chimeric mouse/human mutant amyloid precursor protein (Mo/HuAPP695swe) and a mutant human presenilin 1 (PS1-dE9) both directed to CNS neurons under the prion protein promoter (available by Jackson Laboratory, http://www.jax.org/strain/005864) were used. Mice were housed at the Paracelsus Medical University Salzburg in groups under standard conditions at a temperature of 22 °C and a 12 h light/dark cycle with ad libitum access to standard food and water. Animal care, handling, genotyping and experiment were approved by local ethical committees (BMWFW-66.019/0032-WF/V/3b/2016).

For this study, 12-months old animals were treated for 28 days with PLX5622 chow. Age-matched non-transgenic mice, derived from the breeding of APP Swedish PS1 dE9 (herein abbreviated as APP-PS1) were used as control animals (WT). All animals were adapted to control chow 2 weeks before introducing the PLX5622 chow. The 4 experimental groups consisted of WT and APP-PS1 mice which received control chow and WT and APP-PS1 mice which received the PLX5622 chow for a total of 28 days (see Fig. 4a).

### Perfusion and tissue sectioning

After 28 days of treatment the mice were anesthetized by intraperitoneal injection of a ketamine (20.5 mg/ml, Richter Pharma), xylazine (5.36 mg/ml, Chanelle) and acepromacine (0.27 mg/ml, VANA GmbH) mixture as previously published [35]. Afterwards their thoracic cavity was opened with an incision caudal to the sternum. Animals were manually perfused through the left ventricle with ice cold HBSS containing 15 mM HEPES (all from Thermofisher) and 0.5% glucose (Sigma) to wash out the blood. Afterwards mice were decapitated and brains were extracted from the skull. One total brain hemisphere was immersed in 4% paraformaldehyde (in 0.1 M sodium phosphate solution, pH = 7.4) at 4 °C for 2 days for fixation before being washed in PBS and transferred into 30% sucrose for cryoprotection. When fully soaked with sucrose brain hemispheres were cut in 40 μm slices on dry ice using a sliding microtome (Leika) dividing one brain hemisphere in representative 10ths of the brain. Sections were stored at − 20 °C in cryoprotectant solution (ethylene glycol, glycerol, 0.1 M phosphate buffer pH 7.4, 1:1:2 by volume).

### Fluorescence immunohistochemistry (IHC)

Fluorescence immunohistochemistry of mouse tissue was performed on free-floating sections as previously described [36, 37]. Antigen retrieval was performed depending on the used primary antibody by steaming the sections for 15–20 min. in citrate buffer (pH = 6.0, Sigma). When we used a primary mouse antibody on mouse tissue we used a mouse on mouse kit (Vector laboratories MOM-Kit BMK-2202, Szabo-Scandic) according to the manufacturer’s instructions. The following primary antibodies were used overnight: goat anti-Iba1 (1:500, Abcam), mouse anti-5-Lox (1:50, BD biosciences), monoclonal and polyclonal rabbit anti-5-Lox (1:100, Abcam), goat anti-FLAP (1:150) and rabbit anti-FLAP (1:100, both Novus Biologicals), chicken anti-GFAP (1:2000, Abcam) and guinea pig anti-NeuN (1:700, Millipore). Sections were extensively washed in PBS and incubated for 3 h at RT in secondary antibodies all diluted 1:1000. The following secondary antibodies were used: donkey anti-goat Alexa Fluor 647, donkey anti-guinea pig Alexa Flour 647, donkey anti-chicken Alexa Fluor 488 (Jackson Immuno Research) and donkey anti-rabbit Alexa Fluor 488, donkey anti-rabbit Alexa Fluor 568 and donkey anti-mouse Alexa Fluor 568 (all Invitrogen/Life Technologies). Nucleus counterstaining was performed with 4′.6′-diamidino-2-phenylindole dihydrochloride hydrate (DAPI 1 mg/mL, 1:2000, Sigma). Tissue sections were additionally treated with 0.2% Sudan Black (Sigma) in 70% ethanol for 2 min to reduce the autofluorescence in tissue from old animals [38]. After this treatment the sections were extensively washed in PBS and mounted onto microscope glass slides (Superfrost Plus, Thermo Scientific). Brain sections were cover slipped semi-dry in ProLong Gold Antifade Mountant (Life technologies).

#### Quantification of Iba1^+^/5-Lox^+^ and Iba1^+^/5-Lox^-^ cell numbers in mouse hippocampus

3 confocal z-stack images at 20x magnification of different hippocampi per animal were taken and the number of Iba1^+^/5-Lox^+^ and Iba1^+^/5-Lox^−^ cells were counted using Fiji (ImageJ 1.52p). The mean of 3 images per animal was calculated. Only cells with cell nucleus and clearly visible cell soma were taken for analysis. Percentages of Iba1^+^/5-Lox^+^ and Iba1^+^/5-Lox^−^ cell numbers in total Iba1^+^ cell counts were calculated (*n* = 5 animals/group).

#### Quantification of percentage (%) 5-lox staining

The overall percentage of 5-Lox staining per image was calculated for 3 confocal z-stack images per mouse, all taken at 20x magnification with exact same microscope settings. Images were transformed to maximum intensity projections and a threshold for 5-Lox signals was manually set. We used the analyze particle tool from Fiji (ImageJ 1.51 h) to analyze the number and area (μm^2^) of 5-Lox positive particles. We calculated the sum of 5-Lox positive particle areas (μm^2^) per image and calculated the percentage (%) of staining in respect to the total area of the image (μm^2^). The mean of 3 images per animal was calculated (*n* = 5 animals/group).

### Human FFPE tissue

For qualitative and quantitative analysis of FLAP and 5-Lox immunoreactivity we used 7 μm hippocampal sections from formalin-fixed paraffin embedded human brain samples which were obtained from the Newcastle Brain Tissue Resource (NBTR) in accordance with Newcastle University ethics board and ethical approval awarded by The Joint Ethics Committee of Newcastle and North Tyneside Health Authority (reference: 08/H0906/136). Used samples are listed in Supplementary Table 1).

Irrespective of clinical diagnoses, all brains underwent neuropathological examination according to a routine protocol that uses standardized neuropathological scoring/grading systems, including neurofibrillary tangle (NFT) Braak staging [39, 40] Consortium to Establish a Registry for Alzheimer’s Disease (CERAD) scores [41] Newcastle/McKeith Criteria for Lewy body disease [42], National Institute on Aging – Alzheimer’s Association (NIA-AA) guidelines [43] and Thal phases of amyloid β deposition [44].

For double staining of human brain tissue an ImmPRESS Duet Double Staining HRP/AP Polymer Kit from Szabo-Scandic was used according to manufacturer’s instructions. In short, after deparaffinization, antigen retrieval and blocking of endogenous peroxidase activity slides were incubated overnight with the following primary antibodies: mouse anti-5-Lox (1:50, BD Biosciences), rabbit anti-Iba1 (1:1000, Abcam) or rabbit anti-FLAP (1:100, Novus Biologicus). For staining slides were first incubated with DAB EqV for 2 min, followed by incubation with Vector Red for 30 min (according to protocol ImmPRESS Duet Double Staining HRP/AP Polymer Kit, Szabo-Scandic).

Immunohistochemical stained human brain sections were analyzed using a Virtual Slide Microscope VS120 with the Olympus VS-ASW.L100 software (both from Olympus).

#### Quantification of Iba1^+^/5-lox^+^ and Iba1^+^/5-lox^−^ cell numbers in human hippocampus tissue

Two images/regions of interest with exact same size were selected from the hippocampal dentate gyrus per patient sample. Iba1^+^/5-Lox^+^ and Iba1^+^/5-Lox^−^ cell numbers were counted using Fiji (ImageJ, version: 2.0.0.-rc-69/1.52p) and the mean of 2 images per patient was calculated. Only cells with cell nucleus and clearly visible cell soma were taken for analysis. Percentages of Iba1^+^/5-Lox^+^ and Iba1^+^/5-Lox^−^ cell numbers in total Iba1^+^ cell counts were calculated and presented as pie charts (*n* = 3–5/group).

### Confocal microscopy and image processing

For imaging the Confocal Laser Scanning Microscopes LSM700 and LSM710 from Zeiss were used and gratefully provided by the microscopy core facility of SCI-TReCS (Spinal Cord Injury and Tissue Regeneration Center Salzburg). Images were taken with the ZEN 2011 SP3 or SP7 (black edition) software (Zeiss). Images were taken as confocal z-stacks using 20x, 40x, 63x oil magnification with 0.5 or 0.6 zoom and combined to merged maximum intensity projections. For qualitative analysis 2–3 animals per group were immunohistologically stained and analyzed. For quantitative analysis 5 animals per group were stained and analyzed. All images were edited and processed with the ZEN 2012 (blue edition) software (version 1.1.2.0) and Microsoft PowerPoint. 3D Render was performed using Imaris Software (version 9.1.2, Bitplane).

### RNA isolation and gene expression analysis

To detect mRNA levels of microglia and the LT signaling pathway in different brain regions of 12-months old mice, the total RNA was extracted from mouse hippocampus and cortex. After manual perfusion, animals were decapitated and the tissue of interest was dissected of one brain hemisphere. Brain samples were immediately transferred to RNA later (Sigma) and stored at − 80 °C. Tissue was homogenized in 1 ml Trizol (TRI®Reagent; Sigma). For phase separation, 150 μl of 1-bromo-3-chloropropane (Sigma) were added, vortexed and centrifuged (15 min. at 12.000×g at 4 °C). After transferring the aqueous phase into a new tube 1 μl GlycoBlue™ (Invitrogen) and 500 μl 2-Propanol p.A. (Millipore) were added and vortexed. To obtain RNA, samples were centrifuged (10 min. at 12000 x g at 4 °C). The pellet was washed with 1 ml 75% ethanol, dried and re-suspended in 30 μl RNase-free water (pre-warmed to 55 °C). cDNA was synthesized using the iScript Reverse Transcription Supermix (Bio-Rad). Quantitative gene expression analyses were performed using TaqMan RT-PCR technology. Technical duplicates containing 10 ng of reverse transcribed RNA were amplified with the GoTAQ Probe qPCR Master Mix (Promega) using a two-step cycling protocol (95 °C for 15 s, 60 °C for 60 s; 40 cycles, Bio-Rad CFX 96 Cycler). The following validated exon-spanning gene expression assays were employed: As housekeepers PSMD4 (Mm.PT.56.13046188) and Heatr3 (Mm.PT.56.8463165; both Integrated DNA Technologies) were used. Quantification analyses were performed with qBase Plus (Biogazelle) using geNorm algorithms for multi-reference gene normalization. Bars are represented as mean with SEM (*n* = 5–8 per group).

Following primers were used: Alox5ap (Mm.PT.58.5140995), Alox5 (Mm.PT.58.30176779), LTC4S (Mm.PT.58.5813470), CysLT1R (Mm.PT.58.55581316), CysLT2R (Mm.PT.58.41658034) and GPR17 (Mm.PT.56a.7204101) all from Integrated DNA Technologies.

### RNA sequencing analysis & bioinformatics

Whole transcriptome analysis was performed by Qiagen Genomic Services from total RNA isolated from hippocampal brain regions of 5 animals per group. Analysis was performed in accordance to the company protocols outlined as follows: The library preparation was done using TruSeq® Stranded mRNA Sample preparation kit (Illumina inc). The starting material (500 ng) of total RNA was mRNA enriched using the oligodT bead system. The isolated mRNA was subsequently fragmented using enzymatic fragmentation. Then first strand synthesis and second strand synthesis were performed and the double stranded cDNA was purified (AMPure XP, Beckman Coulter). The cDNA was end repaired, 3′ adenylated and Illumina sequencing adaptors ligated onto the fragments ends, and the library was purified (AMPure XP). The mRNA stranded libraries were pre-amplified with PCR and purified (AMPure XP). The libraries size distribution was validated and quality inspected on a Bioanalyzer 2100 or BioAnalyzer 4200 tapeStation (Agilent Technologies). High quality libraries are pooled based in equimolar concentrations based on the Bioanalyzer Smear Analysis tool (Agilent Technologies). The library pool(s) were quantified using qPCR and optimal concentration of the library pool used to generate the clusters on the surface of a flowcell before sequencing on a NextSeq500) instrument (50 cycles) according to the manufacturer instructions (Illumina Inc.). Quality control of raw sequencing data was conducted using FastQC tool [45]. Reads were then mapped to the genome (*Mus musculus* genome GRCm38) using bowtie2 (version 2.2.2, [46]. Reads that overlap with genes were then counted using HTSEQ tool (version 0.11.2, [47], −m intersection-nonempty -s no -i gene_id -t exon). Expression values of protein coding genes were first normalized and differential expression analysis between the different groups was conducted using Deseq2 [48]. Genes were considered significantly differentially transcribed with an adjusted *p*-value < 0.05 (Benjamini & Hochberg multiple testing correction). Genes were annotated using biomaRt package [49].

### Statistics

For statistical analysis the Prism 5–8 software (GraphPad) was used. The data were tested for normal distribution with the Kolmogorov-Smirnov test. For comparison of two groups an unpaired t test was performed. For comparison of more than two groups, one-way analysis of variance (ANOVA) was used with Tukey’s or Bonferroni’s multiple comparison test as a post-hoc test. The data were depicted as mean and standard error of the mean (SEM) or standard deviation (SD) with a 95% confidence interval as indicated in the respective figure legends. *P* values of *p* < 0.0001 and *p* < 0.001 were considered extremely significant (**** or ***), *p* < 0.01 very significant (**) and *p* < 0.05 significant (*).

## Results

### Cell-type specific 5-Lox and FLAP expression in AD and AD transgenic mouse brains

**Fig. 2:**
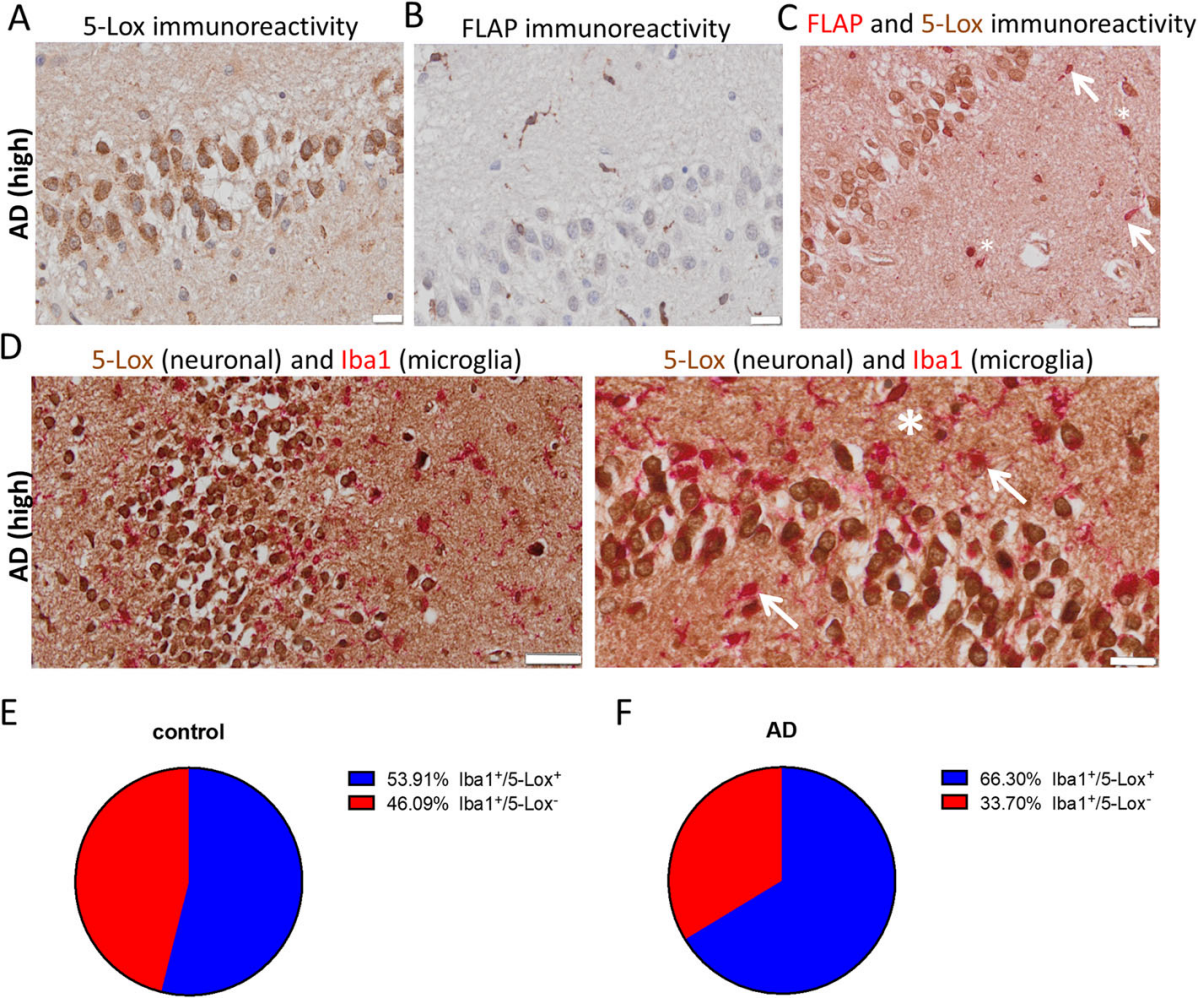
Immunohistochemical analysis for 5-Lox and FLAP expression in human AD brain. **a** 5-Lox immunoreactivity (using monoclonal 5-Lox antibody from BD Biosciences #610694) in human post-mortem AD (high, Braak 6) hippocampus specimen. 5-Lox staining was prominent in cells of the granular cell layer in the dentate gyrus. **b** FLAP was expressed in cells with glial cell morphology but not in neurons. **c** Double staining of 5-Lox with FLAP revealed only minor co-expression of 5-Lox in FLAP positive cells (arrows). Asterisks indicate double positive cells. **d** Double staining of 5-Lox with microglia marker Iba1 showed that non-neuronal 5-Lox expression is associated to some microglia cells (asterisk). Arrows indicate microglia without 5-Lox expression. **e** Percentage of Iba1^+^/5-Lox^+^ and Iba1^+^/5-Lox^−^ cells in Iba1^+^ cells from control and AD (**f**) hippocampus sections. Hematoxylin (**a**, **b**) was used as nucleus stain. Scale: 20 μm (**a**-**c**, **d** right image), 50 μm (**d**, left image)

LTs play a pivotal role in AD pathology, yet it is unclear which cell types are involved in LT biosynthesis in the brain. We performed immunohistochemical analysis for the key enzyme 5-Lox and its activating protein FLAP in brains from human AD patients and age-matched non-pathological controls (see Supplementary Table 1 for clinical and pathological characterization) (Fig. 2). In the hippocampus 5-Lox was predominantly detected in neurons of the granular layer of the dentate gyrus (Fig. 2a) as well as in CA1–3 regions (data not shown). Additionally, we observed low 5-Lox immunoreactivity in some cells, outside of the granular layer, which were according to their morphology most likely of glial identity. In contrast, FLAP was found specifically in non-neuronal cells with glial appearance (Fig. 2b) and was not observed in the granular cell neurons of the dentate gyrus (also not in CA1–3 brain regions - data not shown). FLAP/5-Lox double staining illustrates that 5-Lox stratifies the FLAP expressing cells into a 5-Lox negative (Fig. 2c, arrows) and a 5-Lox positive (Fig. 2c, asterisks) subpopulation. Anticipating that the non-neuronal 5-Lox positive cells might be microglia, we performed double staining of 5-Lox with Iba1, a classical microglia marker (Fig. 2d). This identified a 5-Lox positive (Fig. 2d, asterisk) and a 5-Lox negative (Fig. 2d, arrows) microglia subpopulation. The quantitative analysis revealed that in non-pathological age-matched controls approximately half of the Iba1^+^ population was positive for 5-Lox immunoreactivity (Fig. 2e), while in AD samples about 2/3 of the microglia were 5-Lox positive (Fig. 2f). This suggests that in the context of AD, more microglia might be in the position to synthesize LTs.

**Fig. 3:**
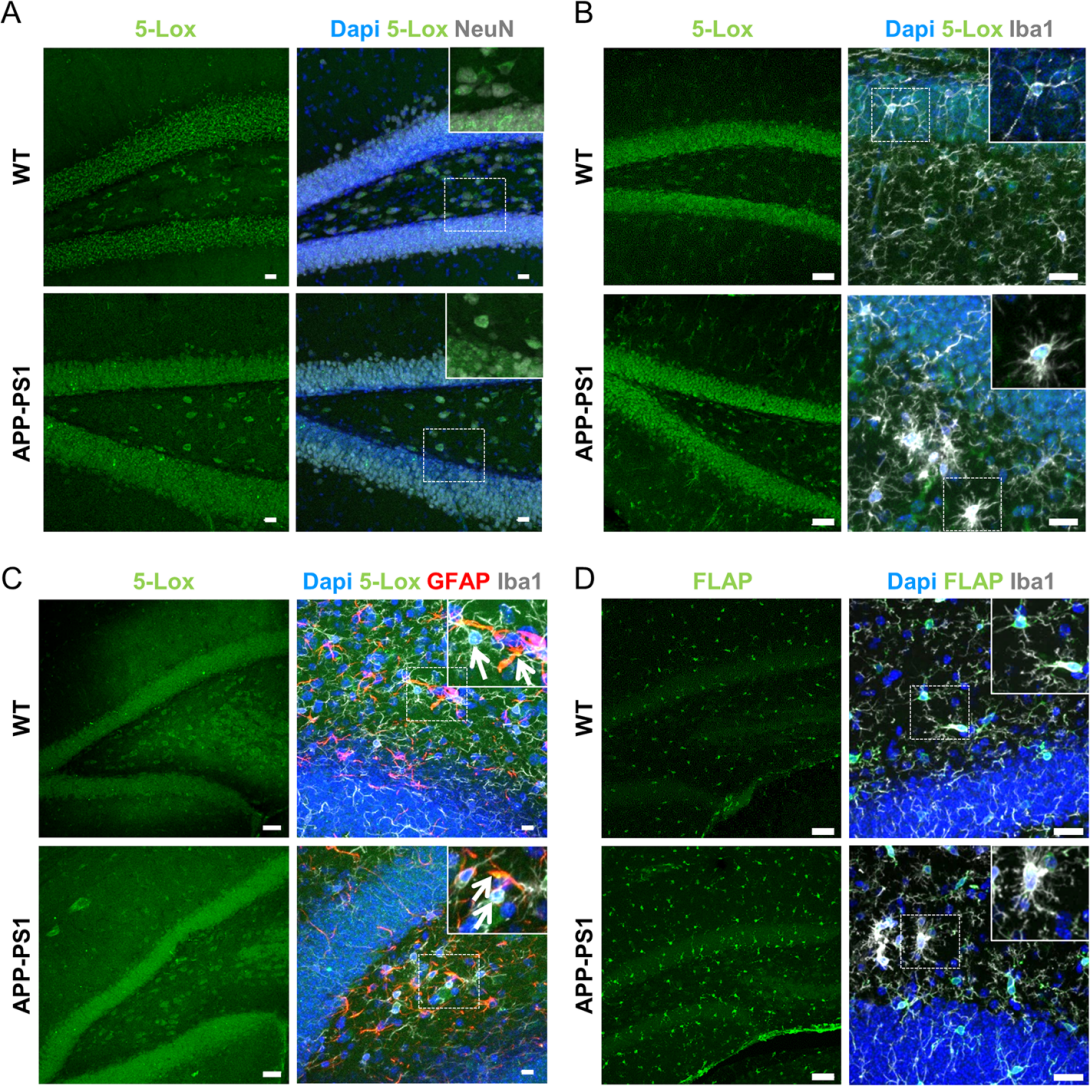
Immunohistochemical analysis for 5-Lox and FLAP expression in WT and APP-PS1 mouse brains. **a** In mouse brains the majority of 5-Lox (green, monoclonal 5-Lox antibody from Abcam #ab169755) immunoreactivity was observed in the granular layer of the dentate gyrus and co-localized with NeuN (white). **b** 5-Lox immunoreactivity (green, using polyclonal 5-Lox antibody from Abcam #ab39347) was also observed outside the granular layer in particular in APP-PS1 mice and co-localized with some Iba1 positive microglia (white) cells of WT and APP-PS1 transgenic mice (inserts). **c** Besides co-expression of 5-Lox (green, using polyclonal 5-Lox antibody from Abcam #ab39347) in some microglia cells, also GFAP (red) positive astrocytes showed immunoreactivity for 5-Lox in WT and APP-PS1 mice (inserts). **d** FLAP (green, polyclonal FLAP antibody from Novus Biologicals #NBP1–84666) was ubiquitously expressed in Iba1 positive microglia cells (white) in WT and APP-PS1 transgenic mice. Dapi (**a**-**d**) was used as nucleus stain. Scale: 20 μm (**b**-**d** right images), 50 μm (**a**-**d** left images)

To corroborate the human data and to stratify our results in a transgenic AD mouse model, we performed immunofluorescence stainings for 5-Lox and FLAP in 13 months old WT and APP-PS1 mice, an age with already advanced pathology (as shown in [36]). We first evaluated several 5-Lox antibodies and their immunoreactivity in neurons and microglia cells (Table 1). As in the human brain specimen, in the granular layer of the dentate gyrus all 5-Lox antibodies showed pre-dominant immunoreactivity in neurons in WT and APP-PS1 mice (example shown in Fig. 3a). While 5-Lox immunoreactivity was more diffuse in the granular layer of WT brain sections, it was dense and more compact in APP-PS1 brains. In addition to the neuronal expression, some Iba1 positive microglia cells (Fig. 3b) as well as GFAP positive astrocytes (Fig. 3c) stained for 5-Lox in WT and APP-PS1 mice. In contrast to the 5-Lox immunoreactivity, but as in the human samples, FLAP staining was confined to microglia (Fig. 3d) and not detected in neurons. We used two different FLAP antibodies from different species (see Table 1) revealing no FLAP immunoreactivity in neurons of the granular cell layer. As all microglia cells were FLAP positive, we can conclude that a microglia subpopulation expresses both FLAP and 5-Lox, particularly observed in APP-PS1 mice. Nevertheless, the vast majority of 5-Lox is predominantly found in neurons.

**Table 1:** Tested 5-Lox and FLAP antibodies on mouse brain tissue and their immunoreactivity in neurons and/or microglia cells. aa = amino acid; − = no, + = mild, ++ = moderate and +++ = strong immunoreactivity

Antibody (host species)	Dilution	Reactivity	Epitope/antigen	Neurons	Microglia	Others
polyclonal Abcam #ab39347 (rabbit)	1:100	mouse, rat, hamster, human, pig	human 5-Lox aa. 130–149	++	++	+
monoclonal Abcam #ab169755 (rabbit)	1:100	mouse, rat, human	human 5-Lox aa. 100–200	++	+	–
monoclonal BD Biosciences #610694 (mouse)	1:50	chicken, human, rat, mouse	human 5-Lox aa. 442–590	+++	–	–
polyclonal Novus Biologicals NBP1–84666 (rabbit)	1:100	human, rat	recombinant protein	–	+++	–
polyclonal Novus Biologicals NB300–891 (goat)	1:150	human, mouse, rat	peptide corresponding to human FLAP (C-terminus)	–	++	–

### Microglia ablation reduced elements of the LT synthesis pathway

**Fig. 4:**
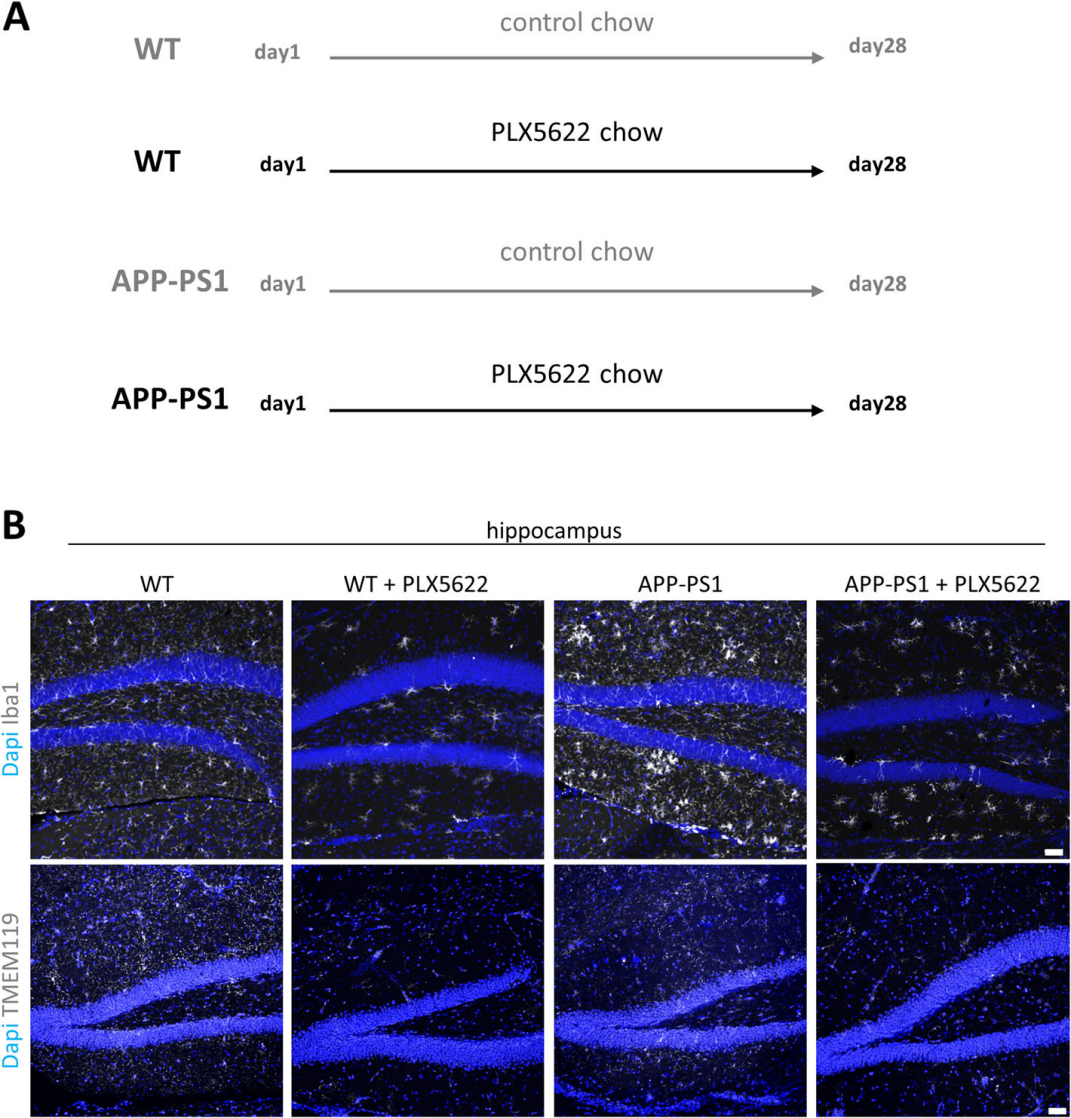
Experimental design of microglia ablation via CSFR1 inhibition. **a** 12 month old mice were treated with the CSF1R inhibitor PLX5622 for a total of 28 days. Treatment was applied in form of control chow or PLX5622 containing chow. **b** WT animals as well as APP-PS1 animals treated with PLX5622 showed a reduction of microglia cells (Iba1^+^ and TMEM119^+^) in the hippocampus compared to the respective control groups. Dapi was used as nucleus stain. Scale: 50 μm (**b**)

To determine the contribution that microglia might play for LT signaling in the brain, we performed a microglia ablation experiment and analyzed its consequences on the LT pathway. 12 months old APP-PS1 transgenic mice with already advanced pathology including increased neuroinflammation were compared to age-matched WT mice. To specifically ablate microglia cells in the brain we used the small molecule compound PLX5622, that is a potent inhibitor of the colony-stimulating factor 1 receptor (CSF1R) [50]. CSF1R inhibitors are widely used to study the role of microglia in several diseases [51–53] including neurodegenerative disease as AD [31, 32, 50, 54], and we and others previously showed that four weeks of treatment with the CSF1R inhibitor PLX5622 depleted microglia in WT and APP-PS1 mice [31, 32, 35]. In this previous experiment, we had treated APP-PS1 and WT mice with either control or PLX5622 chow for a total of 28 days (Fig. 4a). The treatment had strongly reduced numbers of Iba1 positive microglia in the hippocampus and cortex of WT and APP-PS1 (Fig. 4b) as we had previously published (total number of Iba1+ cells in hippocampus: WT 78.63 ± 11.67, WT + PLX5622 13.88 ± 8.25, APP-PS1 145.63 ± 27.21, APP-PS1 + PLX5622 43.63 ± 17.59; cortex: WT 68.71 ± 10.01, WT + PLX5622 5.42 ± 4.79, APP-PS1 141.04 ± 23.77, APP-PS1 + PLX5622 45.46 ± 14.65) [35]. To further confirm microglia ablation, we also used the recently identified microglia specific marker TMEM119 [55]. Indeed, TMEM119 immunoreactivity was diminished in PLX5622 treated animals (Fig. 4b). Similar findings were observed in the cortex (Supplementary Figure 1). A small fraction of Iba1 positive cells with altered cell morphology survived the 4 weeks of PLX5622 treatment, especially in the brains of APP-PS1 mice, as we and others had already previously described [32, 35].

**Fig. 5:**
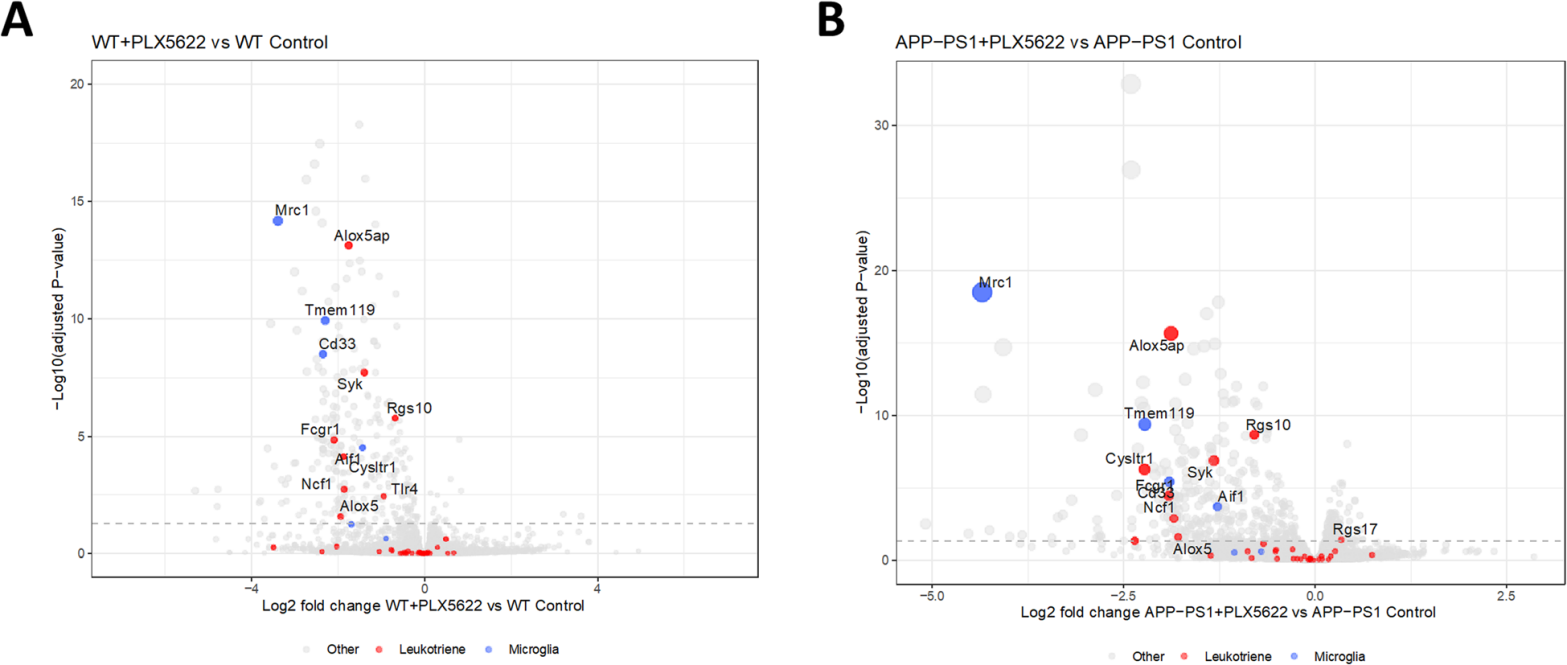
Hippocampal transcriptome analysis revealed significantly downregulated microglia genes and downregulated LT signaling related genes in PLX5622 treated mice. **a** Volcano blots of WT + PLX5622 vs. WT Control and APP-PS1 + PLX5622 vs. APP-PS1 Control (**b**) comparisons illustrating representative microglia genes (*Mrc1*, *TMEM119*, *Cd33*, *Aif1*) that were significantly downregulated (adjusted *p*-value < 0.05) upon microglia cell ablation. Surprisingly, genes involved in the LT signaling pathway (*Alox5ap*, *Alox5*, *Cysltr1*, *Syk*, *Fcgr1*, *Tlr4*, *Ncf1*, *Rgs10*) were significantly downregulated in the hippocampus of WT and APP-PS1 mice upon PLX5622 treatment. Blue spots indicate microglia genes; red spots are LTs related genes and grey spots represent all other genes

After 28 days of PLX5622 treatment total RNA was isolated from the hippocampus and further processed to RNA sequence analysis (RNAseq). The transcriptomic data showed that microglia genes such as *Mrc1* (=CD206), *TMEM119*, *CD33* and *Aif1* (=Iba1) were significantly downregulated after PLX5622 treatment in WT and APP-PS1 animals (Fig. 5, Tables 2 and 3) confirming the microglia ablation at the transcriptome level. Most interestingly in the context of the present study, microglia ablation affected a variety of genes related to LT signaling in WT (Fig. 5a) and APP-PS1 mice (Fig. 5b). Indeed, the majority of LT-related genes were less expressed upon microglia depletion. For example, expression of the *Alox5ap* gene (=FLAP, on protein level) was significantly lower in the microglia depleted brains of WT as well as APP-PS1 animals. The genes *Lta4h* and *Ltc4s*, both enzymes involved in further metabolizing LTA_4_ (see Fig. 1), were not differentially expressed upon microglia ablation. However, *Cysltr1*, the gene for the Cys-LT receptor 1, was significantly decreased upon microglia ablation in WT and APP-PS1 animals. The genes of the two other cysteinyl-LT receptors (*Cysltr2*, *Gpr17*) remained unchanged. Surprisingly, although 5-Lox was found predominantly in neurons, *Alox5* (=5-Lox, on protein level) mRNA expression was lower in the microglia ablated brains (Tables 2 and 3).

**Table 2:** List of differentially expressed genes in the hippocampus of WT + PLX5622 vs. WT control animals: Representative microglia genes and genes involved in the LT signaling pathway were significantly downregulated in WT + PLX5622 mice. Genes with a significant adjusted *p*-value (*p* < 0.05) are presented in bold

WT + PLX5622 vs. WT control			
microglia genes			
**gene**	**gene description**	**fold change**	**adjusted p-value (*p*** **< 0.05)**
***Mrc1***	**mannose receptor, C type 1**	**−3.38**	**6.93E-15**
***Tmem119***	**transmembrane protein 119**	**−2.29**	**1.25E-10**
*Trem2*	triggering receptor expressed on myeloid cells 2	−1.68	5.83E-02
*Cd68*	CD68 antigen	−0.88	2.38E-01
***Cd33***	**CD33 antigen**	**−2.35**	**3.26E-09**
***Aif1***	**allograft inflammatory factor 1**	**−1.42**	**3.20E-05**
leukotrienes signaling pathway			
**gene**	**gene description**	**fold change**	**adjusted p-value (*p*** **< 0.05)**
***Alox5ap***	**arachidonate 5-lipoxygenase activating protein**	**−1.75**	**7.70E-14**
***Alox5***	**arachidonate 5-lipoxygenase**	**−1.94**	**2.68E-02**
*Lta4h*	leukotriene A4 hydrolase	−0.02	9.92E-01
*Ltc4s*	leukotriene C4 synthase	−0.50	9.87E-01
***Cysltr1***	**cysteinyl leukotriene receptor 1**	**−1.87**	**7.59E-05**
*Cysltr2*	cysteinyl leukotriene receptor 2	−0.74	7.77E-01
*Gpr17*	G protein-coupled receptor 17	−0.12	9.07E-01
***Syk***	**spleen tyrosine kinase**	**−1.39**	**1.98E-08**
***Fcgr1***	**Fc receptor, IgG, high affinity I**	**−2.08**	**1.48E−05**
***Tlr4***	**toll-like receptor 4**	**-0.94**	**3.78E-03**
***Ncf1***	**neutrophil cytosolic factor 1**	**−1.85**	**1.92E−03**
***Rgs10***	**regulator of G-protein signalling 10**	**−0.67**	**1.75E-06**
*Rgs17*	regulator of G-protein signaling 17	-0.10	9.48E-01

**Table 3:** List of differentially expressed genes in the hippocampus of APPPS1 + PLX5622 vs. APP-PS1 control animals: Representative microglia genes and genes involved in the LT signaling pathway were significantly downregulated in APP-PS1 + PLX5622 mice. Genes with a significant adjusted *p*-value (*p* < 0.05) are presented in bold

APP-PS1 + PLX5622 vs. APP-PS1 control			
microglia genes			
**gene**	**gene description**	**fold change**	**adjusted p-value (p < 0.05)**
***Mrc1***	**mannose receptor, C type 1**	**−4.34**	**3.51E-19**
***Tmem119***	**transmembrane protein 119**	**−2.22**	**4.47E-10**
*Trem2*	triggering receptor expressed on myeloid cells 2	−1.05	3.26E-01
*Cd68*	CD68 antigen	−0.70	2.86E-01
***Cd33***	**CD33 antigen**	**−1.90**	**4.04E-06**
***Aif1***	**allograft inflammatory factor 1**	**−1.27**	**2.12E-04**
leukotrienes signaling pathway			
**gene**	**gene description**	**fold change**	**adjusted p-value (p < 0.05)**
***Alox5ap***	**arachidonate 5-lipoxygenase activating protein**	**−1.78**	**2.81E-02**
***Alox5***	**arachidonate 5-lipoxygenase**	**−1.88**	**2.44E-16**
*Lta4h*	leukotriene A4 hydrolase	0.09	5.62E-01
*Ltc4s*	leukotriene C4 synthase	−1.36	5.05E-01
***Cysltr1***	**cysteinyl leukotriene receptor 1**	**−2.23**	**5.48E-07**
*Cysltr2*	cysteinyl leukotriene receptor 2	0.75	4.50E-01
*Gpr17*	G protein-coupled receptor 17	−0.13	6.06E-01
***Syk***	**spleen tyrosine kinase**	**−1.32**	**1.44E-07**
***Fcgr1***	**Fc receptor, IgG, high affinity I**	**−1.91**	**3.87E−05**
*Tlr4*	toll-like receptor 4	-0.67	7.90E-02
***Ncf1***	**neutrophil cytosolic factor 1**	**−1.84**	**1.41E−03**
***Rgs10***	**regulator of G-protein signalling 10**	**-0.79**	**2.37E-09**
***Rgs17***	**regulator of G-protein signaling 17**	**0.34**	**4.33E-02**

**Fig. 6:**
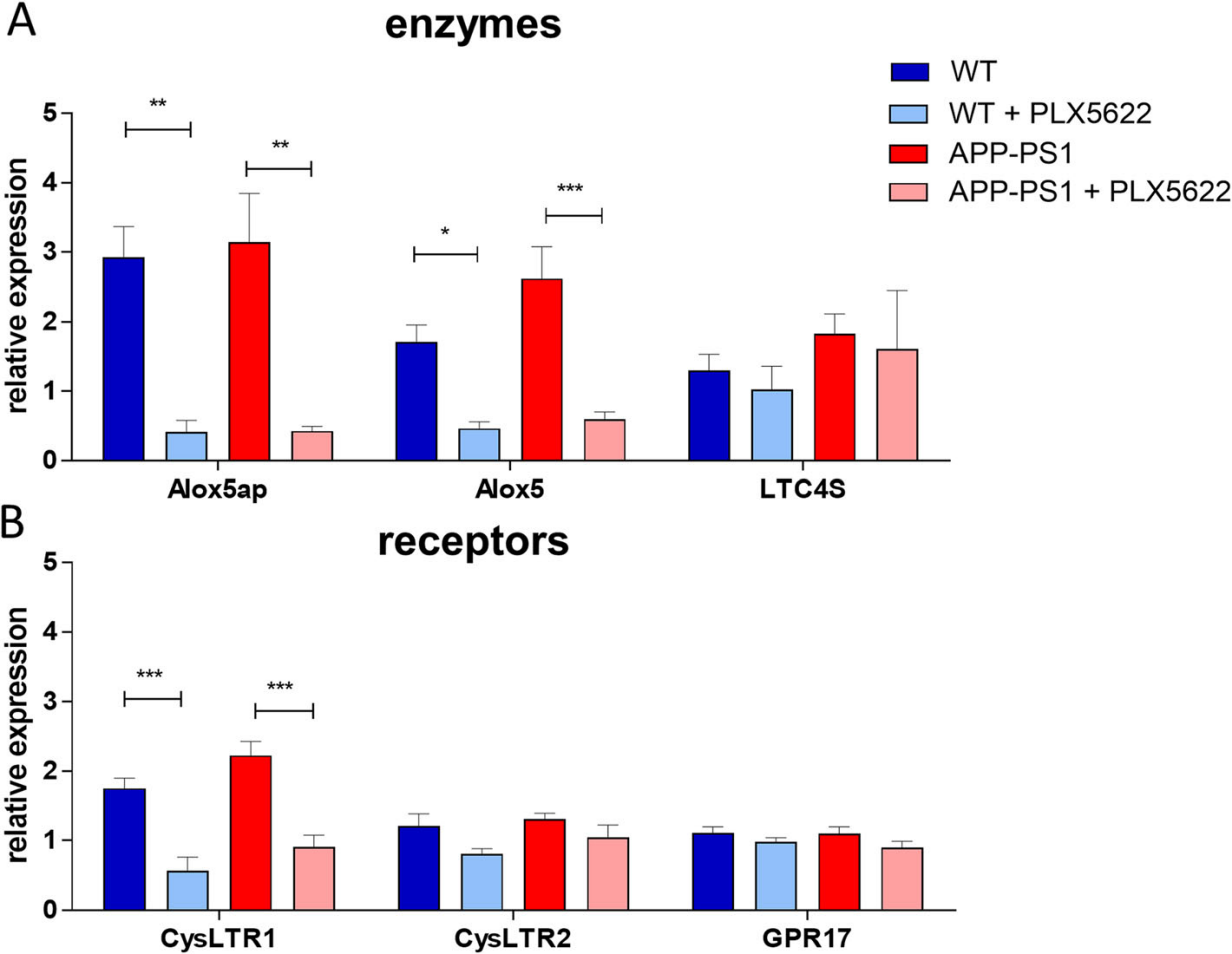
qPCR validation of hippocampal mRNA expression for LT synthesis related genes: **a** Microglia ablation in WT and APP-PS1 mice resulted in significantly lower mRNA expression of *Alox5ap* and *Alox5*. **b** Expression of cysteinyl-LT receptor *CysLTR1* was significantly decreased upon microglia ablation in WT and APP-PS1 mice. One-way analysis of variance with Bonferroni’s multiple comparison test was used. *P*-values < 0.05 were considered significant. Data are shown as mean with SEM

For validation of the RNA sequencing data we performed qPCR gene expression analysis of key components of the LT signaling pathway (Fig. 6a, b). Microglia ablation resulted in a significant decrease in mRNA levels of the gene *Alox5* and *Alox5ap* in WT as well as in APP-PS1 animals (Fig. 6a). On the receptor level, the qPCR data confirmed reduced mRNA expression of *Cysltr1* but not *Cysltr2* or *GPR17* in the hippocampus of microglia depleted brains (Fig. 6b). Similar results were obtained in the cortex (Supplementary Figure 2). Additionally, in the cortex, *Ltc4s* was significantly decreased in APP-PS1 + PLX522 and strongly reduced in WT + PLX5622 animals (Supplementary Figure 2A). In summary, microglia depletion not only diminished expression of *Alox5ap, Ltc4s* (in the cortex) and the receptor *Cysltr1*, but also expression of the *Alox5* gene, which was surprising as the latter is predominantly expressed in neurons.

### FLAP is specifically expressed in microglia cells and abolished upon microglia ablation

**Fig. 7:**
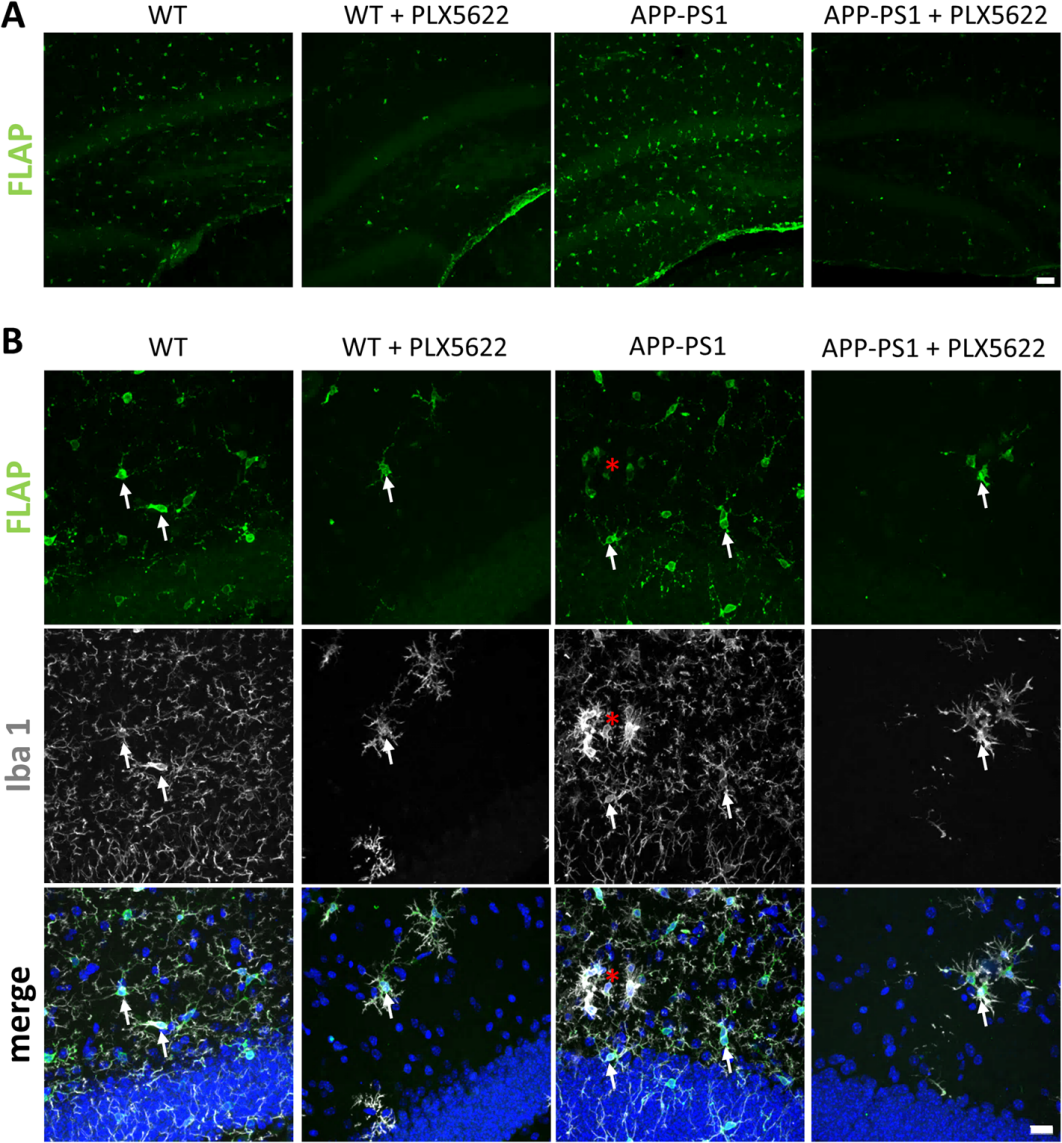
Immunohistochemical analysis of FLAP expression in the hippocampus. **a** Overall FLAP (green, polyclonal FLAP antibody from Novus Biologicals NBP1–84666) expression was more prominent in APP-PS1 compared to WT control animals and highly reduced with PLX5622 treatment. **b** FLAP co-localized with Iba1 (white) positive cells in all groups (arrows). Most interestingly, the intensity of FLAP staining in microglia at sites of amyloid plaques was reduced (red asterisk). Dapi was used as nucleus stain. Scale: 50 μm (**a**), 20 μm (**b**)

To further support the microglia specific expression of FLAP, its expression in the hippocampus in WT and APP-PS1 mice, and its response to the microglia depletion, we performed detailed immunohistochemical analysis (Fig. 7) in the brains of WT and APP-PS1 mice. Overall, in the hippocampus FLAP immunoreactivity was highest in APP-PS1 mice compared to WT and drastically reduced upon microglia ablation in WT and APP-PS1 mice (Fig. 7a). FLAP immunoreactivity was found in Iba1 positive microglia in WT and APP-PS1 mice (Fig. 7b, arrow), similarly to human FLAP staining that showed immunoreactivity in cells with glial morphology (Fig. 2b). Amyloid plaque associated microglia showed detectable but low FLAP immunoreactivity (Fig. 7b, red asterisk), while plaque-distant microglia were strongly FLAP positive (Fig. 7b). FLAP and Iba1 staining were highly diminished in the PLX5622 treated WT and APP-PS1 mice compared to control groups. FLAP was still expressed in treatment resistant Iba1 positive cells. Similar findings were observed in the cortex (Supplementary Figure 3).

### Increased numbers of 5-Lox^+^ microglia in APP-PS1 mice and reduced 5-Lox immunoreactivity after microglia ablation in WT mice

**Fig. 8:**
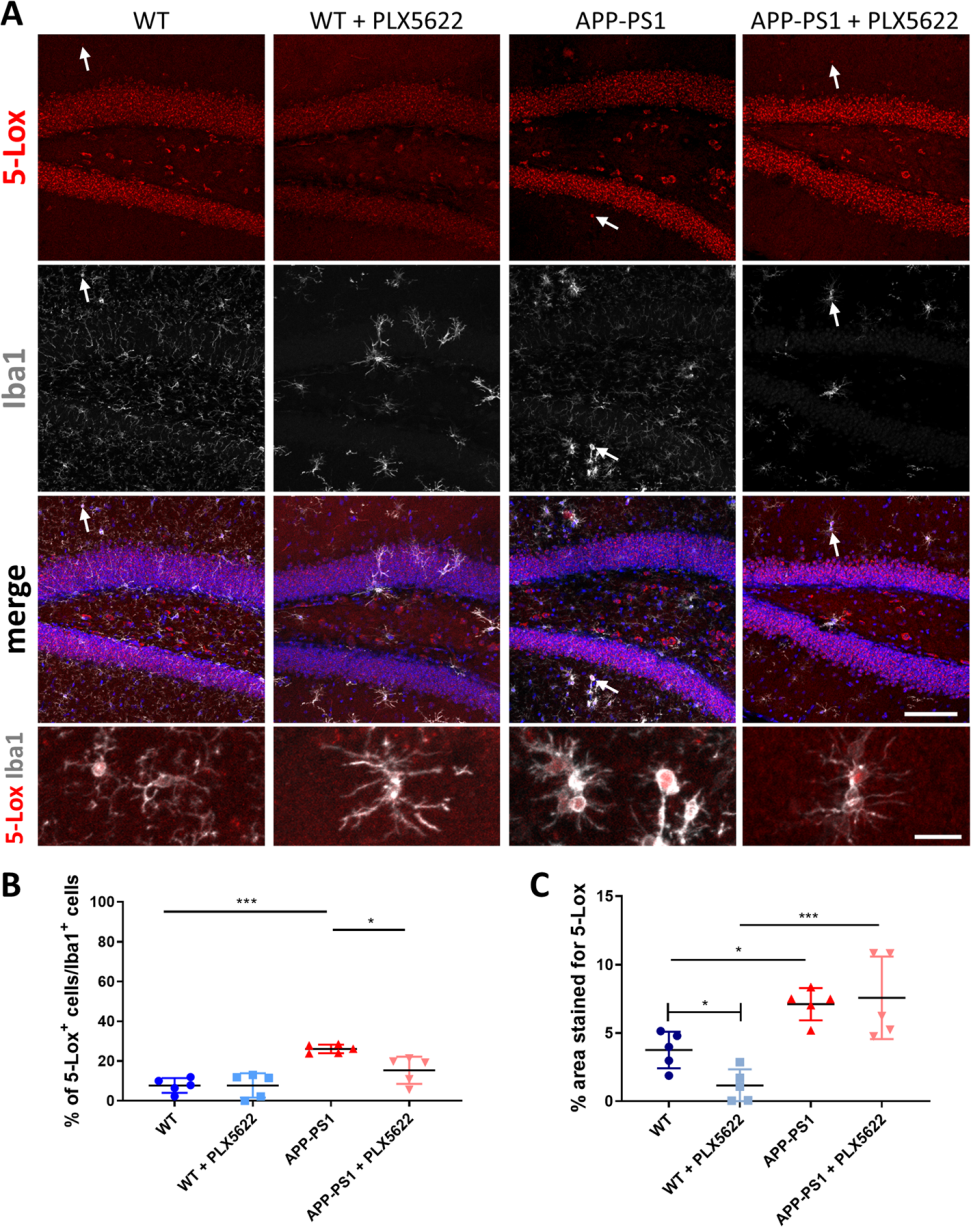
Immunohistochemical analysis of 5-Lox expression in the hippocampus. **a** 5-Lox (red, monoclonal 5-Lox antibody from Abcam #ab169755) was primarily expressed in neurons of the granular cell layer but did also co-localize with some Iba1 (white) positive cells (arrow or inserts). **b** We analyzed the percentage (%) of 5-Lox^+^ cells in the Iba1^+^ cell population revealing increased numbers of 5-Lox + microglia in APP-PS1 mice compared to WT animals. 5-Lox^+^ microglia numbers were reduced in APP-PS1 + PLX5622 animals. **c** The overall 5-Lox staining (% area stained by 5-Lox) was increased in APP-PS1 mice compared to WT animals and reduced upon microglia depletion in WT + PLX5622 mice. Remaining microglia in PLX5622 treated mice displayed altered cell morphology. One-way analysis of variance with Tukey’s multiple comparison test (**b**, **c**) and unpaired t test comparing WT vs. WT + PLX5622 (**c**) was used. P-values < 0.05 were considered significant. Data are shown as mean with SD. Dapi was used as nucleus stain. Scale: 100 μm and 20 μm inserts (**a**)

Next, we analyzed the consequences of microglia ablation on 5-Lox expressing microglia and on overall 5-Lox immunoreactivity in the hippocampus (Fig. 8) and/or cortex (Supplementary Figure 4). We first quantified the percentage of 5-Lox^+^ cells in the Iba1^+^ cell population in the hippocampus of all experimental groups (Fig. 8a, b). In WT animals the percentage of 5-Lox^+^/Iba1^+^ cells was low, but in APP-PS1 mice significantly more Iba1^+^ cells expressed 5-Lox (20–30%). Microglia depletion with PLX5622 did not change the percentage of 5-Lox^+^/Iba1^+^ cells in WT mice but reduced numbers of microglia expressing 5-Lox were detected in APP-PS1 animals (Fig. 8b). This data suggests that microglia react with increased numbers of 5-Lox^+^/Iba1^+^ cells to a chronic inflammatory environment in the APP-PS1 mice. After microglia ablation in APP-PS1 mice, remaining microglia still express 5-Lox, however in a more physiological role comparable to WT animals.

Most interestingly, we observed that upon PLX5622 treatment the overall immunoreactivity of neuronal 5-Lox expression in the granular layer was remarkably reduced in some animals (Fig. 8, Supplementary Figure 5 and Supplementary Figure 7). To verify this observation, we analyzed the percentage of overall 5-Lox staining in the hippocampus (Fig. 8a, c). We observed significantly increased 5-Lox staining (% area) in the hippocampus of APP-PS1 mice compared to WT animals and very surprisingly the percentage of 5-Lox staining was reduced in microglia depleted WT but not in APP-PS1 mice (Fig. 8c). However, within APP-PS1 + PLX5622 mice, a huge inter-individual variation of 5-Lox expression was observed. Significantly reduced 5-Lox expression was particularly seen in in WT + PLX5622 mice. This might indicate a potential feedback and neuronal response to the lack of physiological microglia cell numbers. Similar findings were observed in the cortex (Supplementary Figure 4).

To further confirm reduced neuronal 5-Lox immunoreactivity, we repeated the staining for 5-Lox i) using the exact same monoclonal antibody as used on the human brain specimen (Supplementary Figure 5 and Supplementary Figure 6) and ii) comparing the result also with a polyclonal 5-Lox antibody from rabbit species (Supplementary Figure 7 and Supplementary Figure 8) (for summary of used antibodies see Table 1). Whereas the mouse monoclonal antibody detected 5-Lox only in neurons, the poly- and monoclonal rabbit antibodies detected 5-Lox also in mouse microglia cells. Upon microglia ablation 5-Lox immunoreactivity, as detected by all three different antibodies, was remarkably diminished in the neurons of the dentate gyrus especially from WT + PLX5622 animals.

**Fig. 9:**
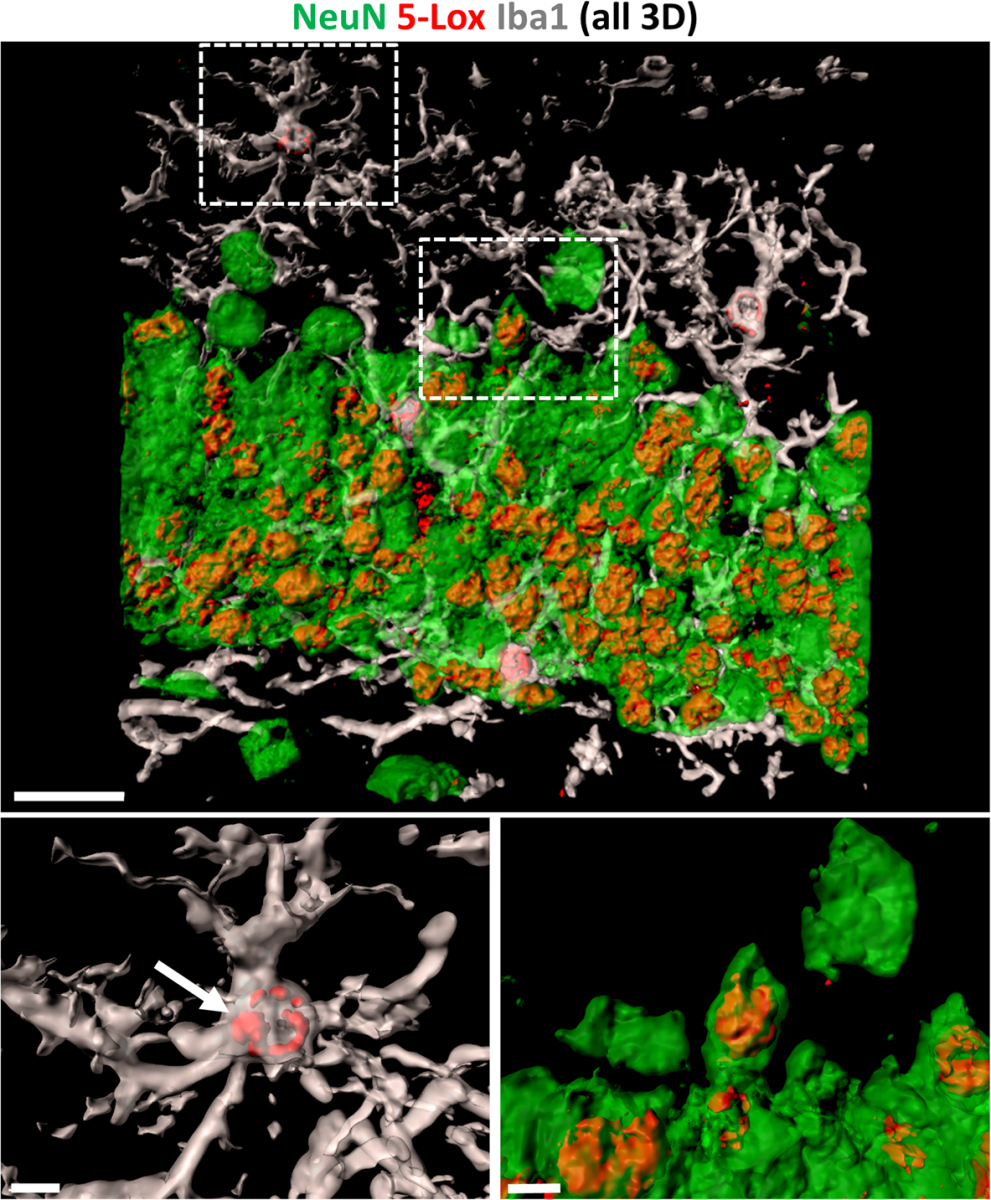
3D Render of images with Imaris Software. 5-Lox immunoreactivity (red, using polyclonal 5-Lox antibody from Abcam #ab39347) is shown in NeuN positive neurons (green) of the granular cell layer in the dentate gyrus and in some Iba1 positive microglia cells (white). In microglia cells 5-Lox staining clustered at the nuclear membrane indicating an activated form of the 5-Lox enzyme (arrow). Scale: 20 μm and 5 μm inserts

**Fig. 10:**
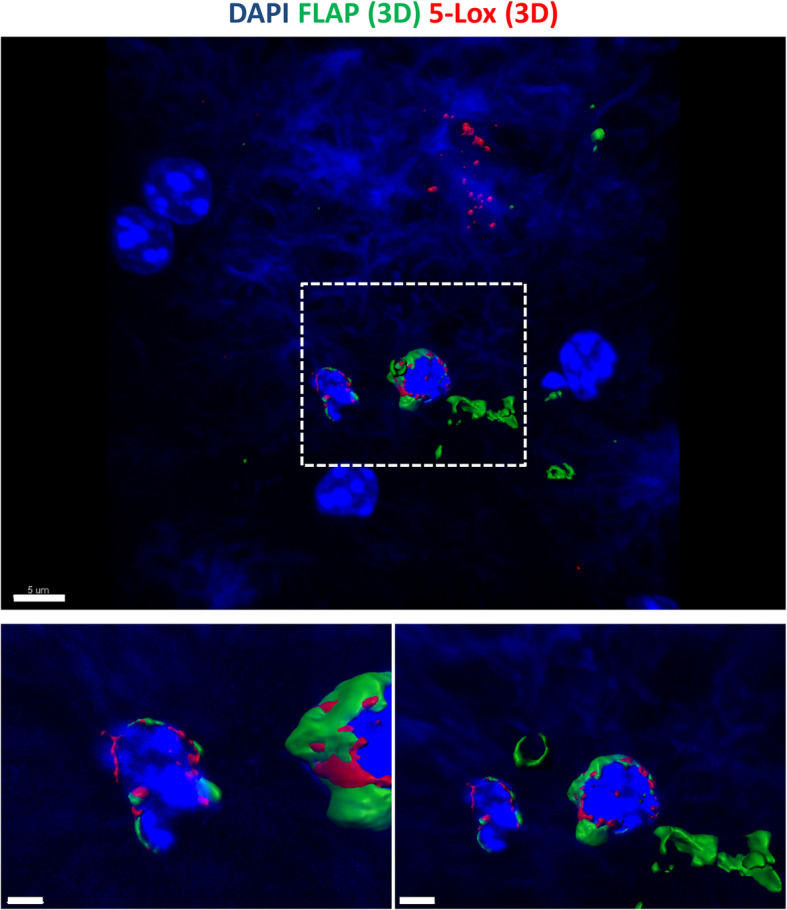
3D Render of images with Imaris Software. 5-Lox (red, using polyclonal 5-Lox antibody from Abcam #ab39347) is expressed in FLAP (green, polyclonal FLAP antibody from Novus Biologicals NB300–891) positive cells associated with the amyloid plaque. 5-Lox and FLAP are in close contact at sites of the nuclear membrane. Dapi was used as nucleus stain. Scale: 5 μm, 1 μm (insert left), 2 μm (insert right)

Visualization of 5-Lox expressing cells in a WT mouse section in high magnification and 3D rendering (Fig. 9) illustrates that 5-Lox is localized in the nucleus of mature neurons but also in a nuclear and perinuclear pattern in some microglia cells (Fig. 9, arrow), see also Video file 1. A huge body of evidence by now indicates that the nuclear envelope is a significant production site of LTs, and that 5-Lox can be found at the nuclear membrane and close to it on both sides of the nuclear membrane (reviewed in [56]; and [57]). Also suggested by the literature [56] the nuclear membrane location of 5-Lox most likely represents an activated form of the 5-Lox enzyme further supporting that microglia might be a production site of LTs, as we find 5-Lox located perinuclear in Iba1+ cells microglia are highly activated at sites of amyloid plaques, so in order to address where FLAP and 5-Lox are expressed intracellular, we performed co-staining of 5-Lox with FLAP in APP-PS1 mice and performed 3D reconstruction of the confocal images (Fig. 10). We observed co-localization of both proteins at sites of the nuclear membrane in cells associated with the amyloid plaque (Fig. 10).

We conclude from this data that FLAP is predominantly expressed by microglia cells, whereas 5-Lox is mainly expressed by neurons and only expressed by a subpopulation of microglia cells. Nevertheless, as FLAP and 5-Lox need to be physically present in the same cell, we assume that microglia are the main source for Leukotrienes in the brain. Furthermore, this illustrates that microglia depletion leads also to a down-regulation of 5-Lox expression in neurons suggesting that the absence of microglia might modulate 5-Lox expression in neurons under healthy conditions. However, to verify this hypothesis and to investigate the contribution of chronic inflammation on neuronal 5-Lox expression further experiments have to be performed in the future.

## Discussion

In the present study we show that FLAP, a key activator molecule of the LT biosynthesis pathway, is expressed in all microglia and not in any other cell type of the brain. 5-Lox is present in a microglia subpopulation, in particular in the context of AD pathology. The most predominant expression of 5-Lox, however, is present in neurons. Microglia ablation drastically reduced the expression of microglia associated LT pathway components such as FLAP and Cysltr1. In addition, and surprisingly, also neuronal 5-Lox expression was diminished after microglia depletion, especially in WT mice. The function of 5-Lox in neurons is unclear, but the absence of FLAP in neurons points towards a 5-Lox function, which might not directly relate to LT synthesis. Nevertheless, microglia clearly influenced 5-Lox expression in neurons, which seemed to depend on the presence of microglia, especially under non-disease conditions. Thus, 5-Lox expression might serve as indicator for the neuroinflammatory stress load in neurons [58]. Alternatively, 5-Lox together with 12-Lox and 15-Lox is also important for the formation of so-called specialized pro-resolving mediators (SPMs), which are anti-inflammatory and pro-resolving lipid mediators that actively promote homeostasis during inflammation [59–61]. A reduction in neuronal 5-Lox following microglia ablation could therefore also be a sign for lesser need of anti-inflammatory stimuli due to a lower numbers of pro-inflammatory signals from microglia. However, the explicit function or functions of 5-Lox in neurons are still elusive and need further investigations.

FLAP is widely expressed in peripheral organs, in particular associated with infiltrating leukocytes, such as in lung [62] and muscle tissue [63]. In the brain, we observed FLAP exclusively expressed in all microglia cells. Interestingly in this context, we did observe a more intense FLAP immunoreactivity in APP-PS1 mice, probably due to an increased number of microglia cells. This presents FLAP as a potential candidate for a novel microglia marker. On the gene expression level, this is supported by a recent study demonstrating that in the brain Alox5ap mRNA expression is microglia-specific as well as significantly differently expressed in AD transgenic mice (TASTPM model) versus WT controls [64]. Furthermore, in these AD mice Alox5ap expression in microglia was age-dependent and highest in the hippocampus and the cortex. In human AD brains Alox5ap expression was especially high in microglia of the temporal cortex as well as in the superior temporal gyrus [64]. This illustrates that FLAP expression underlies dynamic changes along age, brain region and disease status. Indeed, microglia are highly heterogeneous in the healthy [65] as well as in the diseased brain [66]. They exist in several most likely overlapping subpopulations as for example disease associated microglia (DAM) [67], Lipid-droplet-accumulating microglia (LDAM) [68] and AD-associated microglia [69]. We made use of publically available gene expression databases for these microglia subpopulations and analyzed for 5-Lox and FLAP expression, respectively the *Alox5* and *Alox5ap* genes. AD-associated microglia have reduced levels of *Alox5ap* as well as *Alox5* RNA compared to WT microglia [69]. Also, in DAMs *Alox5ap* mRNA expression is lower compared to homeostatic microglia [67]. However, LDAM microglia were not associated with altered *Alox5* or *Alox5ap* levels [68]. Here, we show that plaque associated microglia in APP-PS1 mice have reduced FLAP immunoreactivity suggesting that such FLAP low and plaque associated microglia might be DAMs and/or AD-associated microglia. Therefore, FLAP intensity could be used as marker to further stratify microglia subpopulations and to characterize microglia phenotypes or activation state. This, however, requires further detailed investigations in future.

The cell-type specific expression of 5-Lox and FLAP in the brain has so far been investigated at the mRNA level by in situ hybridization of rat brains in one other study concluding that 5-Lox and FLAP are expressed in neurons [30]. In the present study, we observed FLAP expression specifically in microglia and not in neurons, using two different commercially available FLAP antibodies. 5-Lox staining was present in neurons and limited to a microglia subpopulation. Obviously, the clear identity of the latter requires further investigation. As our results are only partially in line with the above mentioned study from 1996 [30], which indicated neuron-specific expression of 5-Lox and FLAP, we intensively researched microglial and neuronal expression of *Alox5ap* and *Alox5* in publically available databases. First, microglia isolated from mouse cerebral cortex express roughly 27 times more *Alox5ap* (FPKM: 321.5) than *Alox5* (FPKM: 12.3) (following FPKM values taken from: http://www.brainrnaseq.org/ [70, 71], suggesting that in microglia FLAP is higher expressed compared to 5-Lox. The same is true for humans (microglia *Alox5ap* (FPKM 140.5), *Alox5* (FPKM 5.9)). Second, in mouse neurons, expression of *Alox5ap* (FPKM 0.8) and of *Alox5* (FPKM 0.1) is very low and also in human neurons *Alox5ap* (FPKM 2.0) and *Alox5* (FPKM 0.1) are expressed at a very low level (data derive from non-disease and young conditions). Third, *Alox5* in mouse microglia (FPKM 12.3) was higher expressed compared to neurons (FPKM 0.1). Similarly, this is the case in humans (*Alox5*: in microglia FPKM 5.9, in neurons FPKM 0.1). Forth, in mice *Alox5ap* was higher expressed in microglia (FPKM 321.5) compared to neurons (FPKM 0.8). The same was true in humans (Alox5ap: in microglia FPKM 140.5, in neurons FPKM 2.0. This is mostly in line with our histological data from mouse hippocampus and cortex where we show wide expression of FLAP in microglia, but only a smaller proportion of microglia express 5-Lox. We observed 5-Lox immunoreactivity predominantly in neurons and moderate levels of *Alox5* in the hippocampus and cortex, although we investigated already aged and diseased animals. Therefore, the comparison with the above-mentioned database is limited and has to be carefully interpreted as data derive from young and healthy animals.

5-Lox and FLAP are the initiators of LTs synthesis [72, 73] and need to be in close proximity within a cell [22]. Thus, we expected a high level of co-expression between 5-Lox and FLAP, as it was previously shown in mast cells for example by single molecule localization microscopy [74]. Apparently, only a subpopulation of microglia co-express 5-Lox and FLAP. A vast majority of 5-Lox is found in neurons. If this neuronal 5-Lox might contribute to LT synthesis is unclear, but since 5-Lox activity requires FLAP for its membrane anchoring and activation, it is unlikely that neuronal 5-Lox contributes to LT synthesis. 5-Lox immunoreactivity has been demonstrated in neurons, and also in astrocytes and microglia after MCAO in rats [75]. Furthermore, increased neuronal 5-Lox staining was also reported in AD patients, showing 5-Lox in the granular layer of the dentate gyrus and CA1–3 regions [1]. The latter paper was drawing also the attention to the fact that different 5-Lox antibodies might show different results, i.e. different glial or non-glial cell 5-Lox immunoreactivity [1]. In the present work we approached the topic on antibody specificity by using three commercially available and different 5-Lox antibodies (Abcam and BD Biosciences) from different hosts (mouse and rabbit) targeting different epitopes, i.e. either the C- or N-terminal sites, of the 5-Lox molecule. Consistently, all three antibodies detected 5-Lox primarily always in neurons, two of the antibodies detected 5-Lox also in microglia (see Table 1).

A possible way of 5-Lox distribution in the CNS could be extracellular vesicles (EVs) and their intercellular exchange might explain the presence of 5-Lox in various CNS cell types. EVs include not only various proteins and mRNA but also nucleic acids and lipids (reviewed in [76]). Furthermore, EVs are described to carry eicosanoids which can be shuttled together with an enzyme machinery for eicosanoid production (reviewed in [77]). For example, EVs from human macrophages and dendritic cells in the context of lung inflammation and/or asthma were shown to comprise FLAP and 5-Lox as well as other components of the LTs biosynthesis pathway [78]. However, if this is also true in the healthy or diseased brain and if microglia and neuron can shuttle 5-Lox via exosomes as response to inflammatory stimuli is at this point pure speculation. Thus, considering the present data and concluding from the available literature, the majority of 5-Lox immunoreactivity is confined to neurons and to a lesser extend it is expressed in some microglia cells. Single cell transcriptome profiling of neurodegenerative brains will certainly be required to further underscore our findings.

## Conclusion

**Fig. 11:**
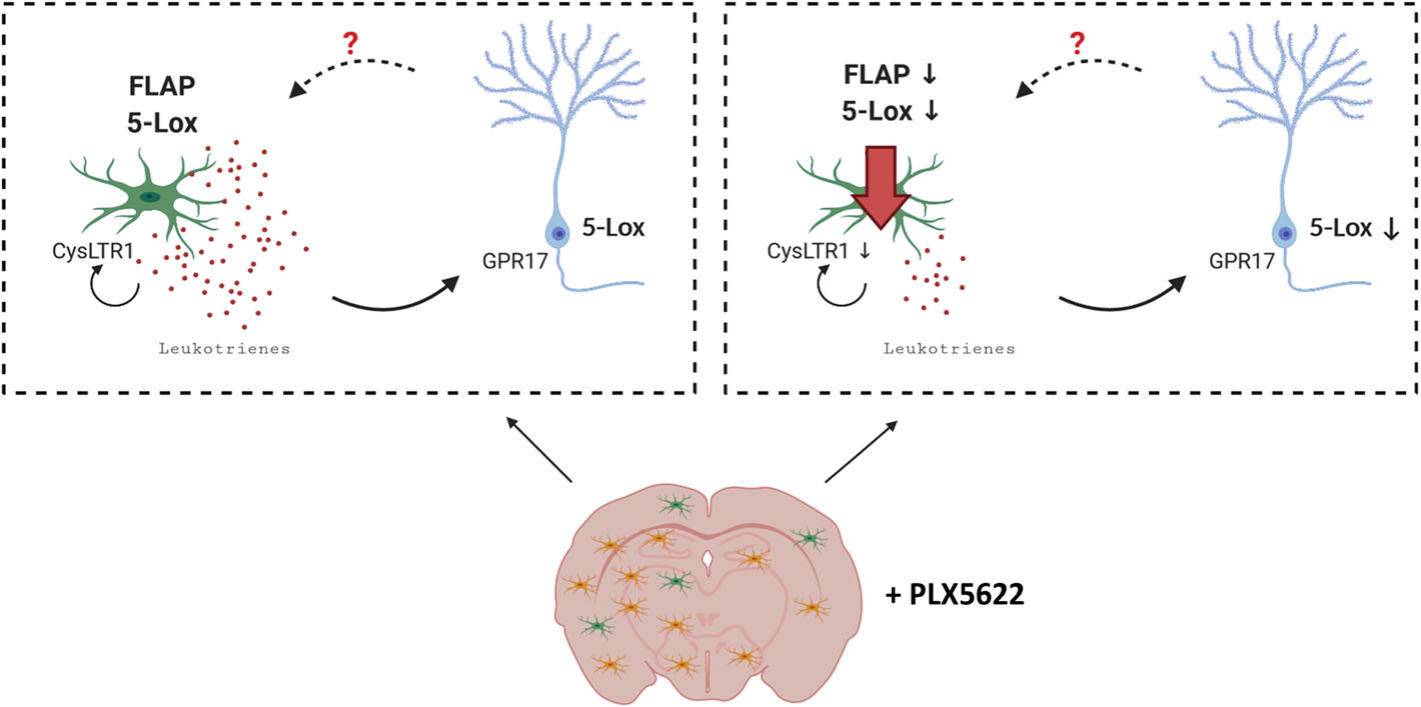
Hypothetical model for microglia as source of LTs in the brain. Key elements of the LTs synthesis pathway are FLAP and 5-Lox, which are co-expressed in some microglia cells (green). Additionally, 5-Lox staining was observed in neurons (blue). It is known that 5-Lox and FLAP protein need to form an intracellular complex to start the synthesis pathway resulting in the production of LTs (left panel). With the herein shown microglia ablation experiment (+PLX5622), we could confirm microglia cells as potential source for LTs. In microglia ablated brains (right panel) FLAP and 5-Lox expression as well as CysLTR1 was highly decreased. Additionally, our data supports the idea of a putative microglia neuron interaction, as 5-Lox immunoreactivity was reduced in neurons of microglia ablated WT brains. LTs thereby possibly affect microglia in an autocrine manner via the CysLTR1, and neurons via GPR17. We hypothesize a putative feedback loop between neurons and microglia involving LTs signaling in the context of neuroinflammation. Image was created with BioRender.com

We conclude that FLAP and 5-Lox co-expression identifies a microglia subpopulation which is the presumed cellular source of LTs in the brain and which is more prominent in the context of AD. FLAP is expressed exclusively and by all microglia in the brain presenting it as a possible novel microglial marker. The majority of 5-Lox is found in neurons and is elevated in neurodegeneration. As neurons do not show detectable FLAP, it is unlikely that they produce LTs unless they have a FLAP-like mechanism to anchor 5-Lox to membranes. Neuronal 5-Lox expression strongly depends on the presence of microglia suggesting the presence of a feedback signal between neurons and microglia. It might be a first response to inflammatory and/or degenerative conditions. LTs might either in an autocrine or paracrine manner stimulate microglia via CysLTR1 [6], which has been localized on microglia, but also signal to neurons via GPR17, a LT receptor predominantly expressed on neurons [79] (Fig. 11). Our herein shown data deliver new insights in the cellular components involved in LT synthesis in the brain, with strong focus on microglia. This data might improve knowledge on the use of already existing anti-LT drugs in diseases of the CNS.

## Supplementary information

**Additional file 1: Supplementary Figure 1.** Immunohistochemistry for Iba1 and TMEM119 in cortical brain regions. Microglia (Iba1^+^ and TMEM119^+^) were ablated in WT and APP-PS1 mice using PLX5622. Dapi was used as nucleus stain. Scale: 50 μm.

**Additional file 2: Supplementary Figure 2.** qPCR of cortical mRNA expression for LT synthesis related genes: (A) Microglia ablation in WT and APP-PS1 mice resulted in significant decline of *Alox5ap* mRNA. *Alox5* and *LTC4S* mRNA expression was significantly decreased after microglia ablation in APP-PS1 animals and reduced in WT mice. (B) Relative mRNA expression of cysteinyl-LT receptor *CysLTR1* was significantly decreased upon microglia ablation in WT and APP-PS1 mice. One-way analysis of variance with Bonferroni’s multiple comparison test was used. *P*-values < 0.05 were considered significant. Data are shown as mean with SEM.

**Additional file 3: Supplementary Figure 3.** Analysis of FLAP expression in the cortex. (A) Overall FLAP (green) expression was more prominent in APP-PS1 compared to WT control animals and highly reduced with PLX5622 treatment. (B) FLAP co-localized with Iba1 (white) positive cells in all groups (arrows). Most interestingly, the intensity of FLAP staining in microglia at sites of amyloid plaques was reduced (red asterisk). Dapi was used as nucleus stain. Scale: 50 μm (A), 20 μm (B).

**Additional file 4: Supplementary Figure 4.** Immunohistochemical analysis of 5-Lox expression in the cortex. We verified 5-Lox immunostaining in neurons and microglia cells using a monoclonal 5-Lox antibody. 5-Lox (red, monoclonal 5-Lox antibody from Abcam #ab169755) was primarily expressed in neurons but did also co-localize with some Iba1 (white) positive cells (arrows and inserts). Most interestingly, overall the 5-Lox staining was reduced upon microglia ablation using CSF1R inhibitor PLX5622. Remaining microglia in PLX5622 treated mice displayed altered cell morphology. Dapi was used as nucleus stain. Scale: 20 μm and 10 μm inserts.

**Additional file 5: Supplementary Figure 5.** To confirm our data on the reduction of 5-Lox staining in the dentate gyrus after microglia ablation, we performed an additional staining with the mouse anti-5-Lox antibody (monoclonal 5-Lox antibody from BD Biosciences #610694), the same used for human tissue. With this antibody 5-Lox immunoreactivity was exclusively found in neurons and did not co-localize with Iba1 positive cells in all groups (inserts). However, most interestingly the 5-Lox staining was again reduced in the granular layer of the dentate gyrus upon microglia ablation. Dapi was used as nucleus stain. Scale: 50 μm and 20 μm inserts.

**Additional file 6: Supplementary Figure 6.** To confirm our data on the reduction of 5-Lox staining in cortical neurons after microglia ablation, we performed an additional staining with the mouse anti-5-Lox antibody (monoclonal 5-Lox antibody from BD Biosciences #610694), the same used for human tissue. With this antibody 5-Lox immunoreactivity was exclusively found in neurons and did not co-localize with Iba1 positive cells in all groups (inserts). However, most interestingly the 5-Lox staining was again reduced in neurons upon microglia ablation. Dapi was used as nucleus stain. Scale: 20 μm (both).

**Additional file 7: Supplementary Figure 7.** Immunohistochemical analysis of 5-Lox expression in the hippocampus. 5-Lox (red, using polyclonal 5-Lox antibody from Abcam #ab39347) was primarily expressed in neurons of the granular cell layer but did also co-localize with some Iba1 (white) positive cells (arrows and inserts). Most interestingly, overall the 5-Lox staining in the granular layer was reduced upon microglia ablation using CSF1R inhibitor PLX5622. Remaining microglia in PLX5622 treated mice displayed altered cell morphology. Dapi was used as nucleus stain. Scale: 20 and 10 μm inserts.

**Additional file 8: Supplementary Figure 8.** Immunohistochemical analysis of 5-Lox expression in the cortex. 5-Lox (red, using polyclonal 5-Lox antibody from Abcam #ab39347) was primarily expressed in neurons but did also co-localize with some Iba1 (white) positive cells (inserts). Most interestingly, overall the 5-Lox staining was reduced upon microglia ablation using CSF1R inhibitor PLX5622. Dapi was used as nucleus stain. Scale: 50 and 20 μm inserts.

**Additional file 9: Supplementary Table 1.** List of patients samples including neuropathological assessment of pathological diagnosis (Path Diag), NFT Braak, CERAD, NIA-RI, Thal Abeta, NIA-AA, Braak LB and McKeith stage scores as wells as age, gender and MMSE score information.

**Additional file 10: Video file 1**. 3D Render of 5-Lox expression in the hippocampus of WT mouse. 5-Lox in red, Iba1 in white and NeuN in green. Video was generated using Imaris software.

## Data Availability

Authors declare availability of data and material upon request.

## Abbreviations

5-Lox: 5-lipoxygenase
AA: Arachidonic acid
AD: Alzheimer’s disease
AIF1: Allograft inflammatory factor 1
Alox5: Arachidonate 5-lipoxygenase (gene name)
Alox5ap: Arachidonate 5-lipoxygenase activating protein (gene name)
APPPS1: APP Swedish PS1 dE9
BBB: Blood-brain-barrier
CA1–3: Cornu Ammonis 1–3
CD33: Cluster of differentiation 33, sialic acid binding Ig-like lectin 3
CNS: Central nervous system
CSF1R: Colony stimulating factor 1
CysLT1R: Cysteinylleukotriene receptor 1
CysLT2R: Cysteinylleukotriene receptor 2
CysLTs: Cysteinylleukotrienes
DAM: Disease associated microglia
DHA: Docosahexaenoic acid
EPA: Eicosapentaenoic acid
EVs: Extracellular vesicles
FLAP: 5-lox activating protein
GPR17: G-protein coupled receptor 17
Iba1: Ionized calcium-binding adapter molecule 1
LAM: Lipid droplet associated microglia
LT: Leukotriene
LTA4: Leukotriene A 4
Lta4h: Leukotriene A 4 hydrolase
LTB4: Leukotriene B 4
LTC4S: Leukotriene C 4 synthase
Mrc1: Mannose receptor C-Type 1
NBTR: Newcastle brain tissue resource
NFT: Neurofibrillary tangles
SPM: Specialized pro-resolving mediator
TMEM119: Transmembrane protein 119
WT: Wild-type
