# Supplementary figures and images for: Microglia depletion diminishes key elements of the leukotriene pathway in the brain of Alzheimer’s Disease mice
Source: Acta Neuropathol Commun. 2020 Aug 8;8:129. doi: 10.1186/s40478-020-00989-4 (PMC7414992; doi:10.1186/s40478-020-00989-4)

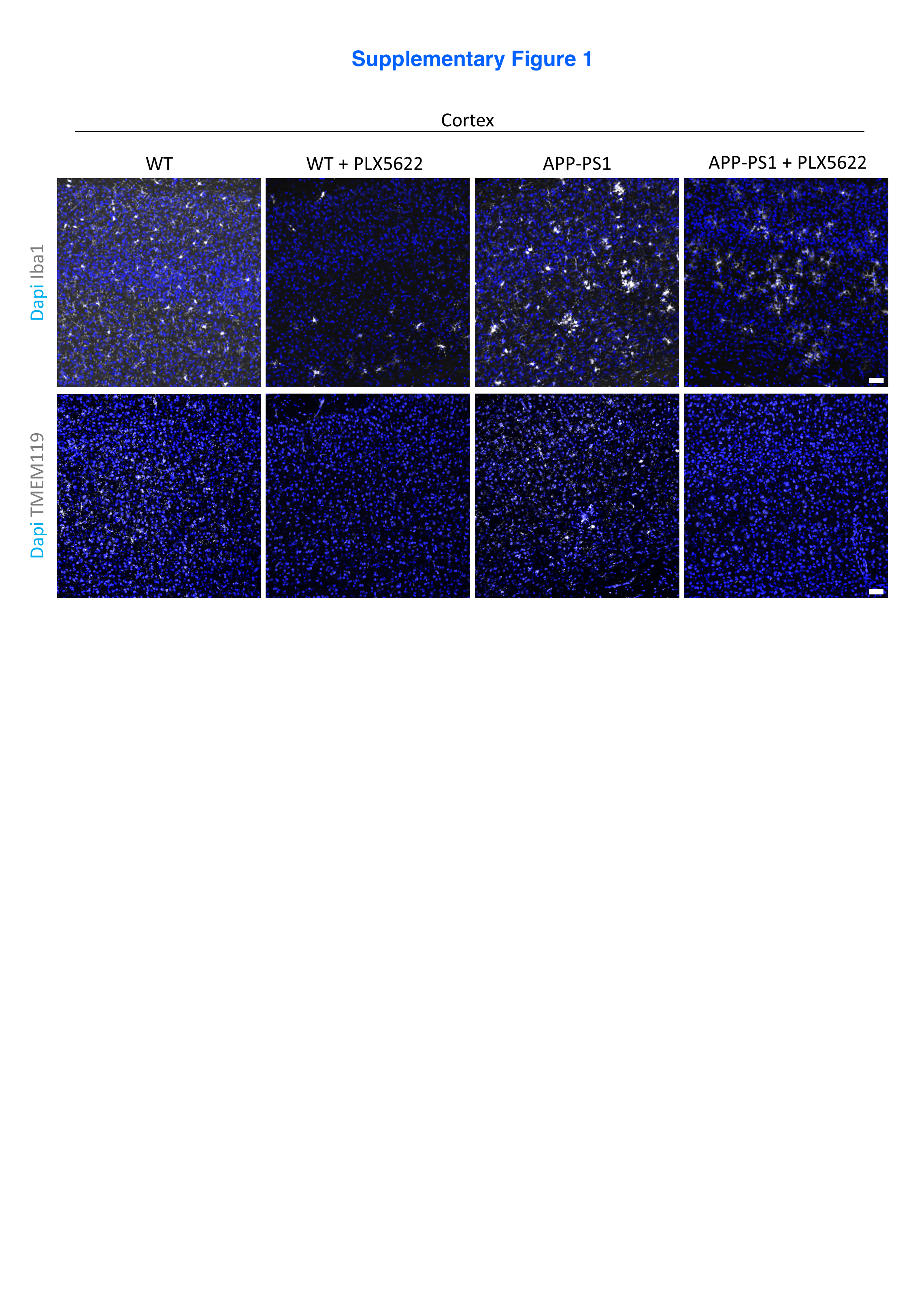

Supplement: Supplementary file 1 — Additional file 1: Supplementary Figure 1. Immunohistochemistry for Iba1 and TMEM119 in cortical brain regions. Microglia (Iba1+ and TMEM119+) were ablated in WT and APP-PS1 mice using PLX5622. Dapi was used as nucleus stain. Scale: 50 μm. [file 40478_2020_989_MOESM1_ESM.tif]

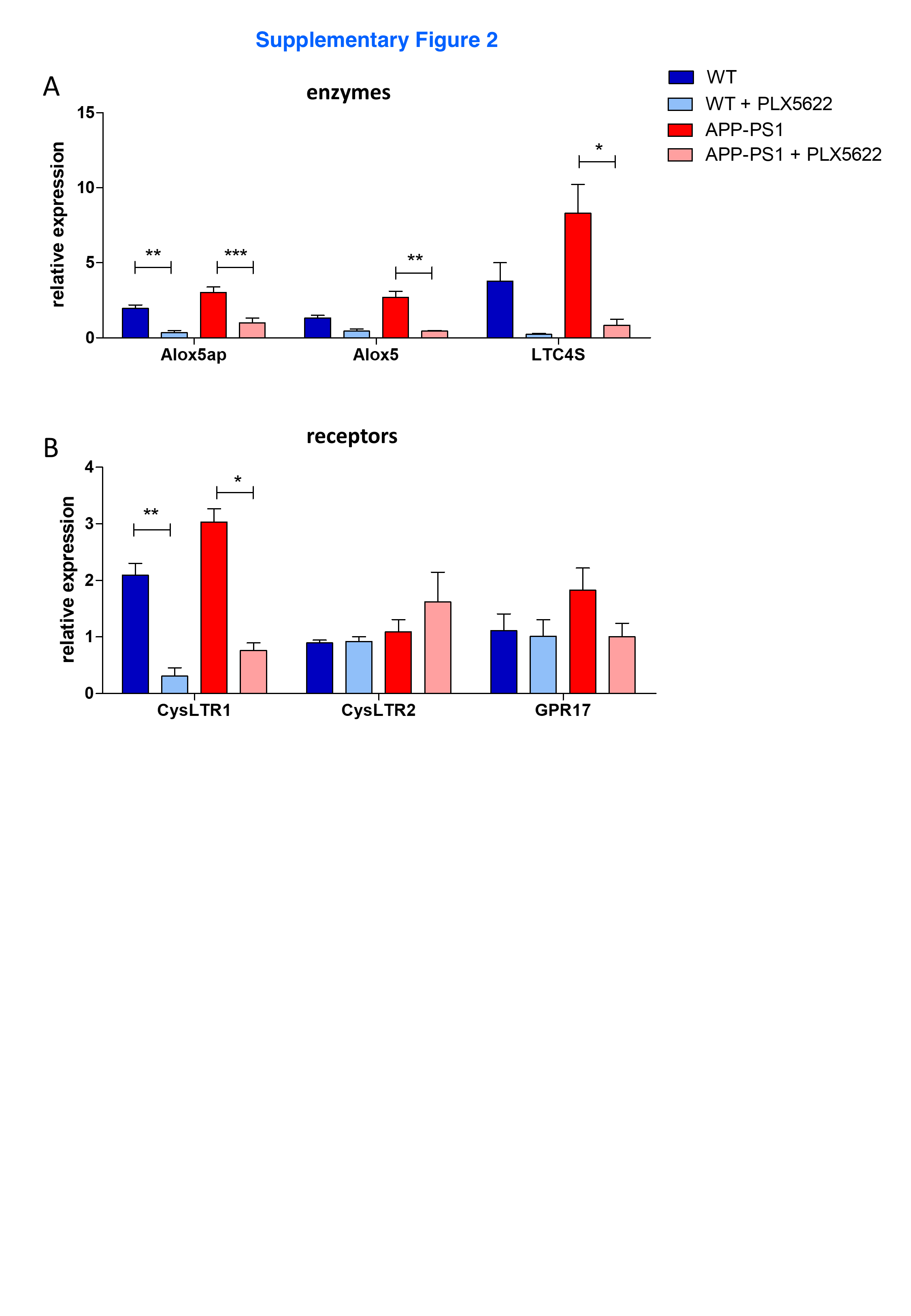

Supplement: Supplementary file 2 — Additional file 2: Supplementary Figure 2. qPCR of cortical mRNA expression for LT synthesis related genes: (A) Microglia ablation in WT and APP-PS1 mice resulted in significant decline of Alox5ap mRNA. Alox5 and LTC4S mRNA expression was significantly decreased after microglia ablation in APP-PS1 animals and reduced in WT mice. (B) Relative mRNA expression of cysteinyl-LT receptor CysLTR1 was significantly decreased upon microglia ablation in WT and APP-PS1 mice. One-way analysis of variance with Bonferroni’s multiple comparison test was used. P-values < 0.05 were considered significant. Data are shown as mean with SEM. [file 40478_2020_989_MOESM2_ESM.tif]

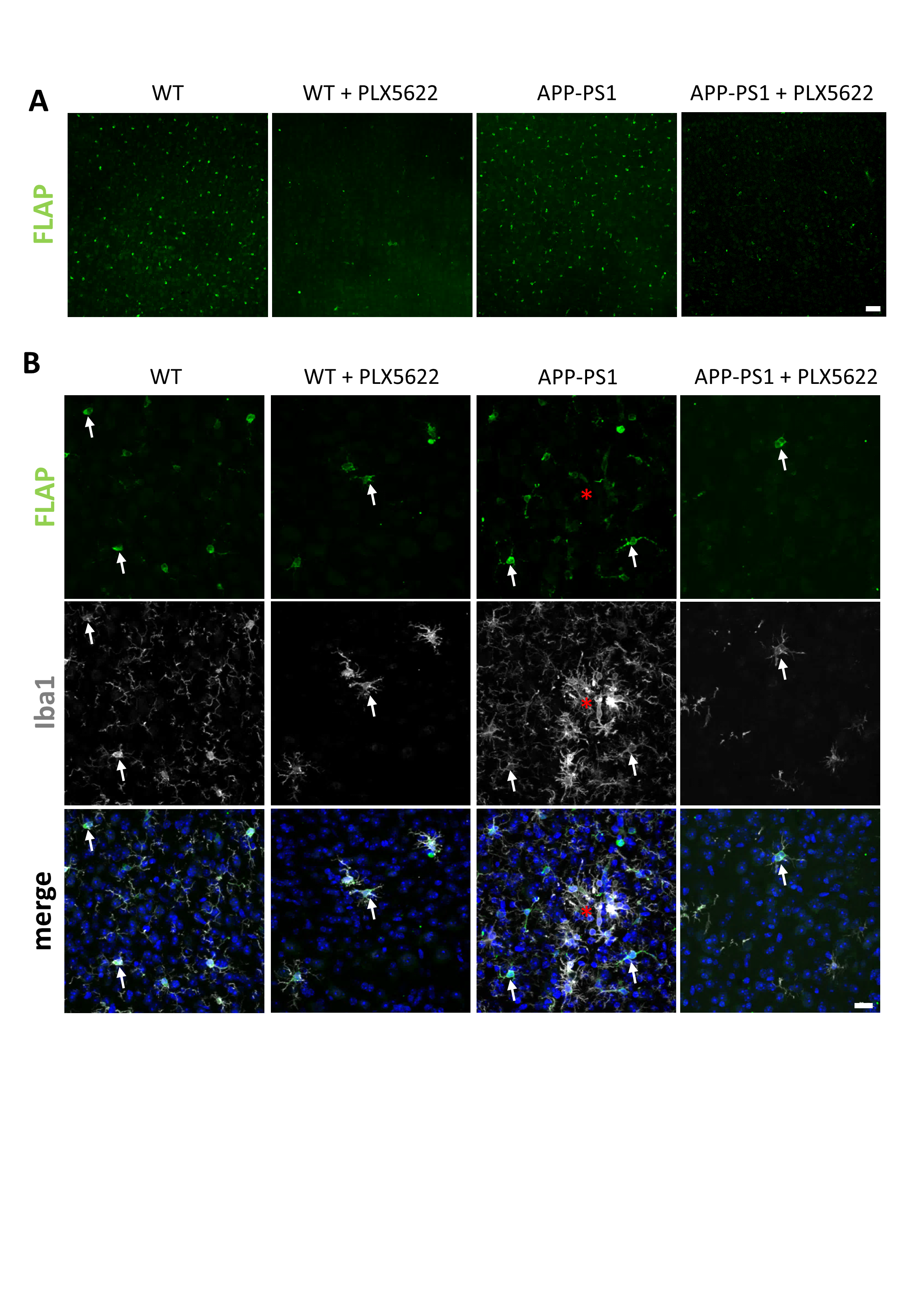

Supplement: Supplementary file 3 — Additional file 3: Supplementary Figure 3. Analysis of FLAP expression in the cortex. (A) Overall FLAP (green) expression was more prominent in APP-PS1 compared to WT control animals and highly reduced with PLX5622 treatment. (B) FLAP co-localized with Iba1 (white) positive cells in all groups (arrows). Most interestingly, the intensity of FLAP staining in microglia at sites of amyloid plaques was reduced (red asterisk). Dapi was used as nucleus stain. Scale: 50 μm (A), 20 μm (B). [file 40478_2020_989_MOESM3_ESM.tif]

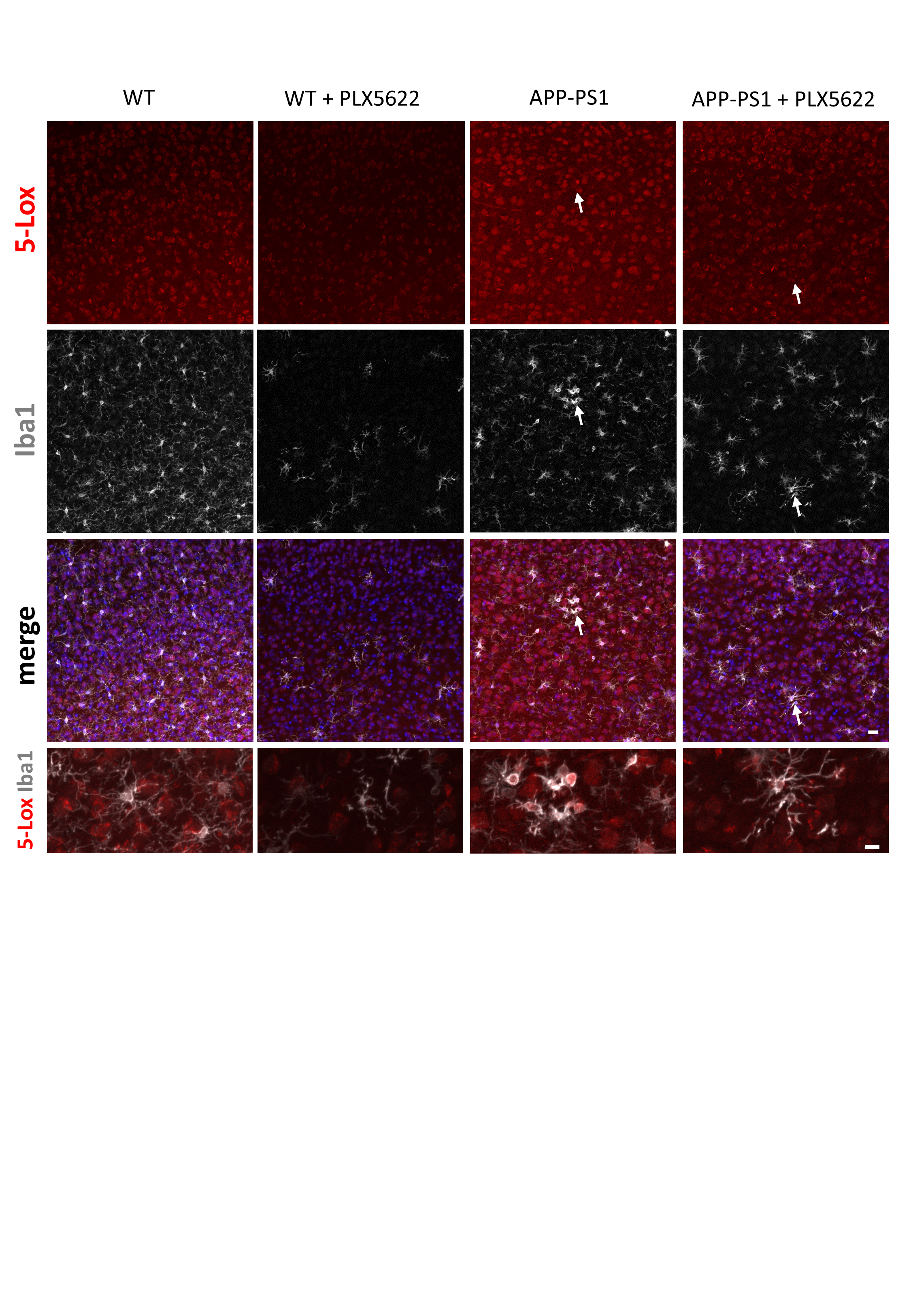

Supplement: Supplementary file 4 — Additional file 4: Supplementary Figure 4. Immunohistochemical analysis of 5-Lox expression in the cortex. We verified 5-Lox immunostaining in neurons and microglia cells using a monoclonal 5-Lox antibody. 5-Lox (red, monoclonal 5-Lox antibody from Abcam #ab169755) was primarily expressed in neurons but did also co-localize with some Iba1 (white) positive cells (arrows and inserts). Most interestingly, overall the 5-Lox staining was reduced upon microglia ablation using CSF1R inhibitor PLX5622. Remaining microglia in PLX5622 treated mice displayed altered cell morphology. Dapi was used as nucleus stain. Scale: 20 μm and 10 μm inserts. [file 40478_2020_989_MOESM4_ESM.tif]

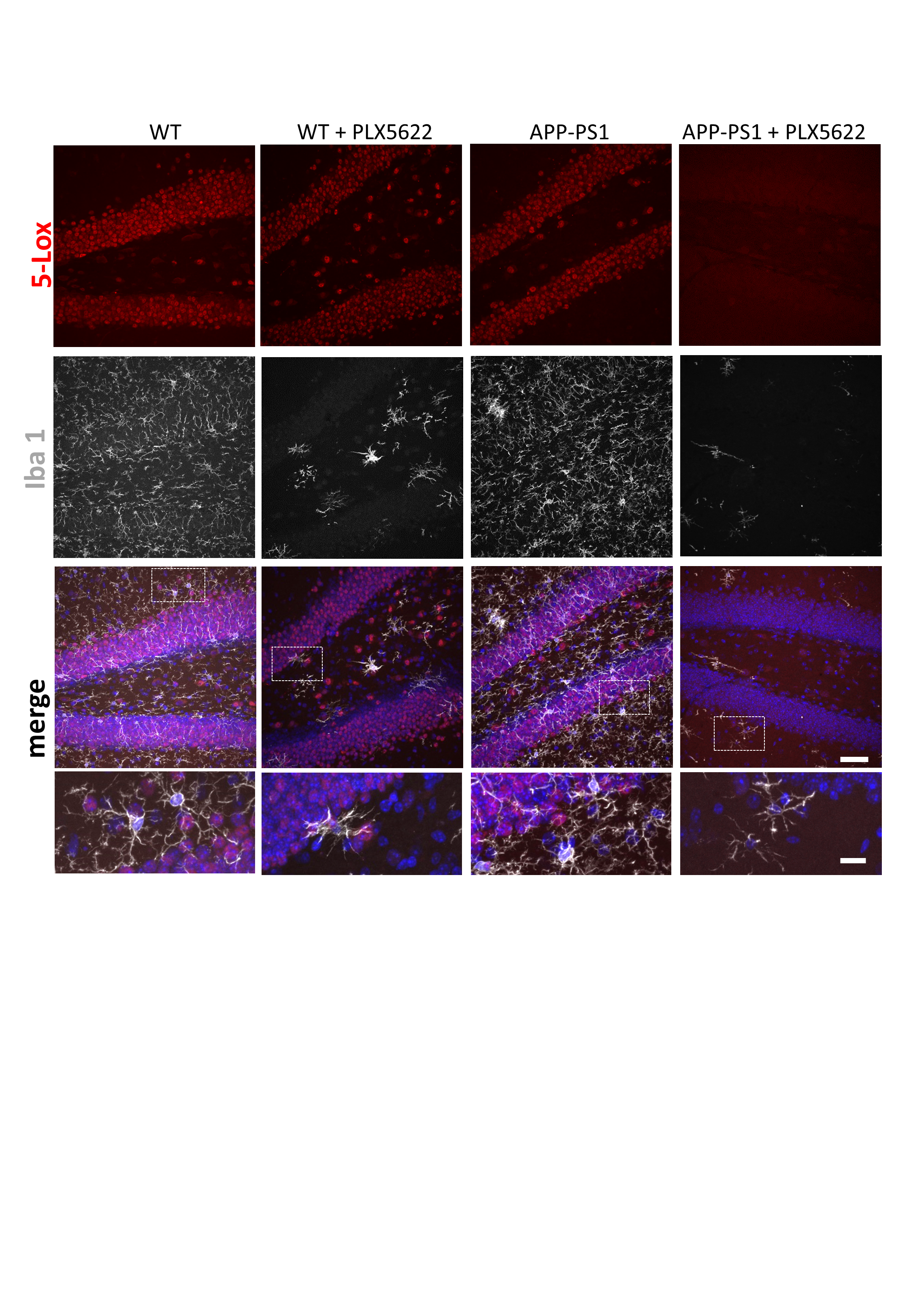

Supplement: Supplementary file 5 — Additional file 5: Supplementary Figure 5. To confirm our data on the reduction of 5-Lox staining in the dentate gyrus after microglia ablation, we performed an additional staining with the mouse anti-5-Lox antibody (monoclonal 5-Lox antibody from BD Biosciences #610694), the same used for human tissue. With this antibody 5-Lox immunoreactivity was exclusively found in neurons and did not co-localize with Iba1 positive cells in all groups (inserts). However, most interestingly the 5-Lox staining was again reduced in the granular layer of the dentate gyrus upon microglia ablation. Dapi was used as nucleus stain. Scale: 50 μm and 20 μm inserts. [file 40478_2020_989_MOESM5_ESM.tif]

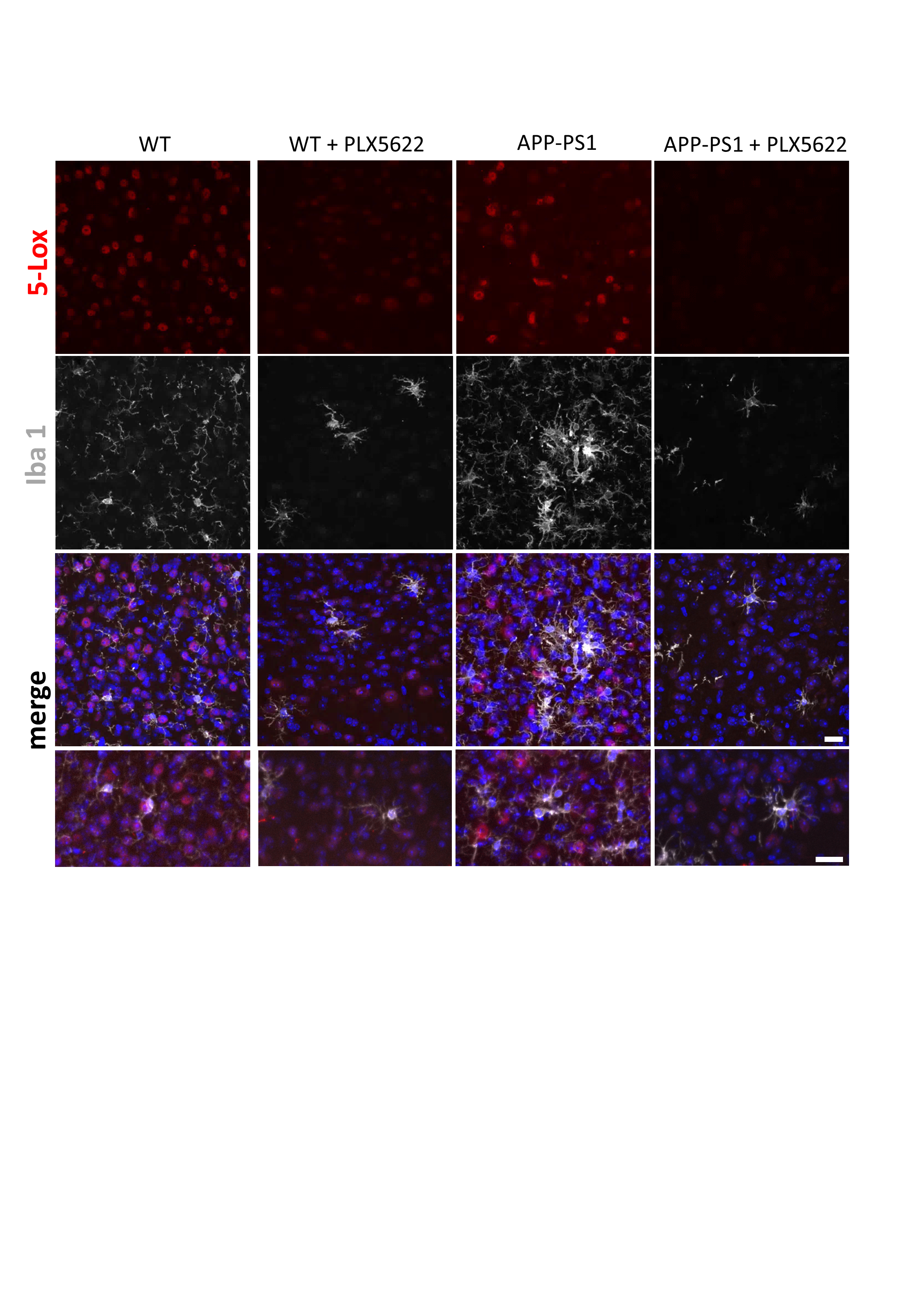

Supplement: Supplementary file 6 — Additional file 6: Supplementary Figure 6. To confirm our data on the reduction of 5-Lox staining in cortical neurons after microglia ablation, we performed an additional staining with the mouse anti-5-Lox antibody (monoclonal 5-Lox antibody from BD Biosciences #610694), the same used for human tissue. With this antibody 5-Lox immunoreactivity was exclusively found in neurons and did not co-localize with Iba1 positive cells in all groups (inserts). However, most interestingly the 5-Lox staining was again reduced in neurons upon microglia ablation. Dapi was used as nucleus stain. Scale: 20 μm (both). [file 40478_2020_989_MOESM6_ESM.tif]

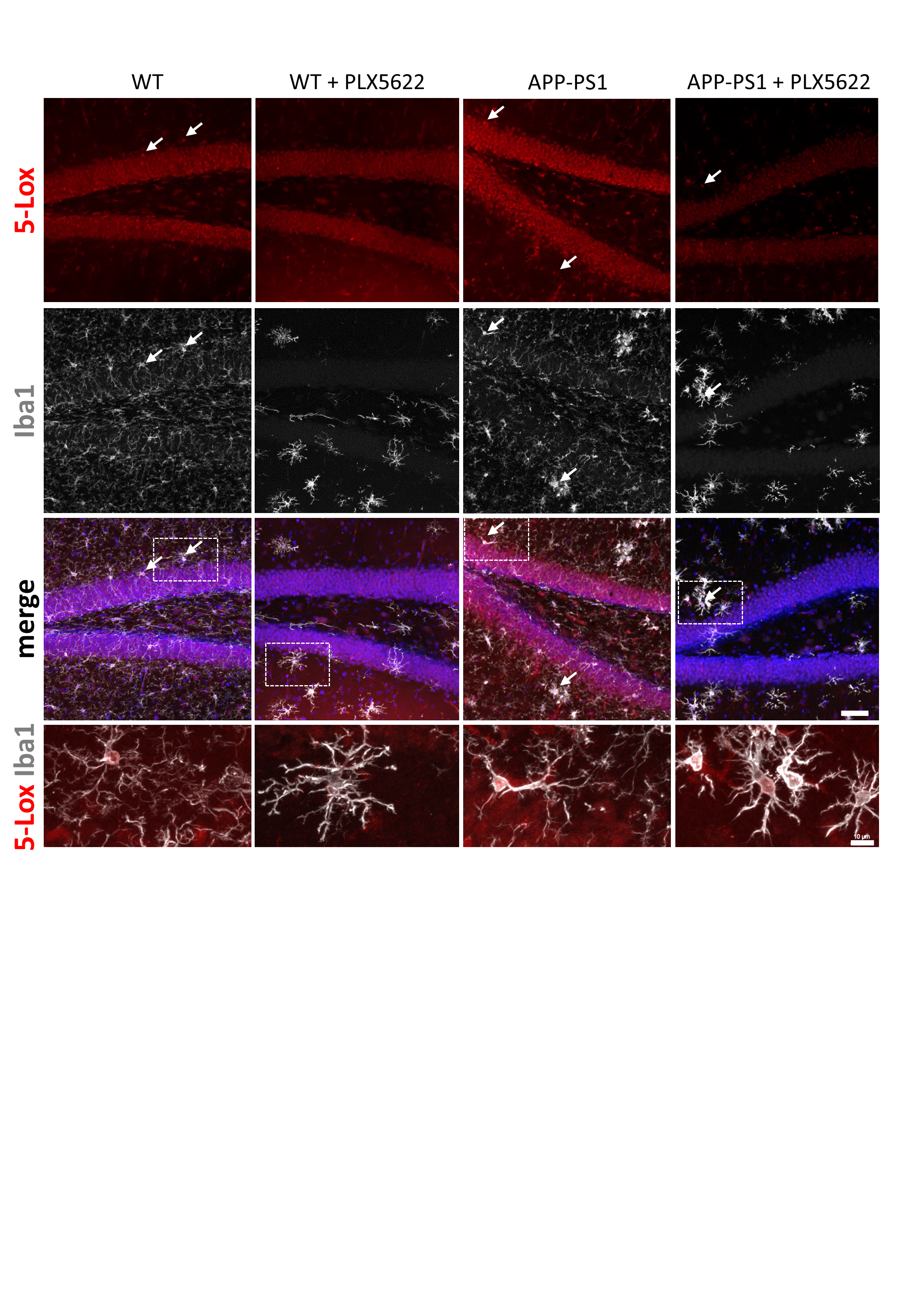

Supplement: Supplementary file 7 — Additional file 7: Supplementary Figure 7. Immunohistochemical analysis of 5-Lox expression in the hippocampus. 5-Lox (red, using polyclonal 5-Lox antibody from Abcam #ab39347) was primarily expressed in neurons of the granular cell layer but did also co-localize with some Iba1 (white) positive cells (arrows and inserts). Most interestingly, overall the 5-Lox staining in the granular layer was reduced upon microglia ablation using CSF1R inhibitor PLX5622. Remaining microglia in PLX5622 treated mice displayed altered cell morphology. Dapi was used as nucleus stain. Scale: 20 and 10 μm inserts. [file 40478_2020_989_MOESM7_ESM.tif]

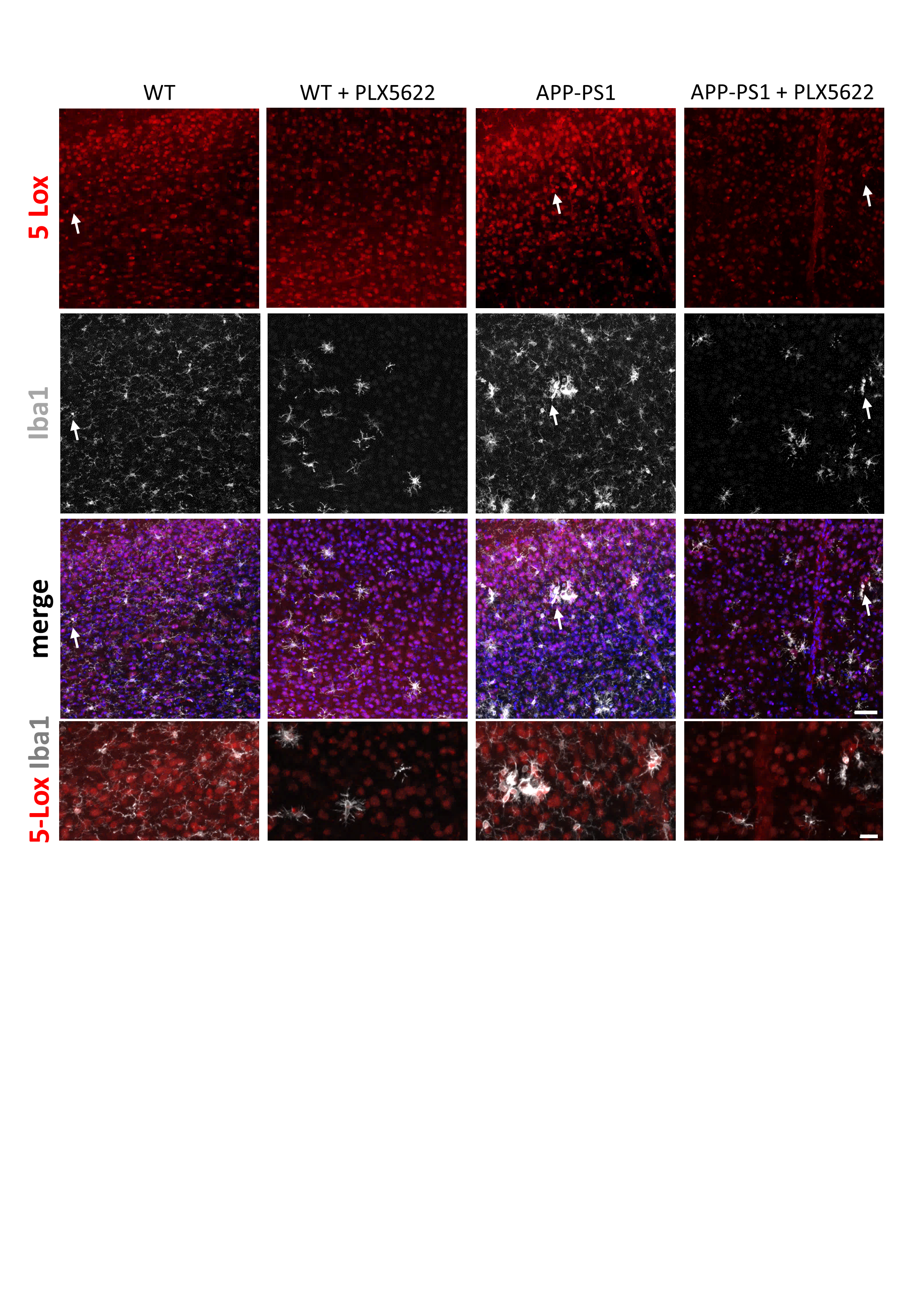

Supplement: Supplementary file 8 — Additional file 8: Supplementary Figure 8. Immunohistochemical analysis of 5-Lox expression in the cortex. 5-Lox (red, using polyclonal 5-Lox antibody from Abcam #ab39347) was primarily expressed in neurons but did also co-localize with some Iba1 (white) positive cells (inserts). Most interestingly, overall the 5-Lox staining was reduced upon microglia ablation using CSF1R inhibitor PLX5622. Dapi was used as nucleus stain. Scale: 50 and 20 μm inserts. [file 40478_2020_989_MOESM8_ESM.tif]
