# Supplementary material for: Microglia depletion diminishes key elements of the leukotriene pathway in the brain of Alzheimer’s Disease mice
Source: Acta Neuropathol Commun. 2020 Aug 8;8:129. doi: 10.1186/s40478-020-00989-4 (PMC7414992; doi:10.1186/s40478-020-00989-4)
Supplement: Supplementary file 9 — Additional file 9: Supplementary Table 1. List of patients samples including neuropathological assessment of pathological diagnosis (Path Diag), NFT Braak, CERAD, NIA-RI, Thal Abeta, NIA-AA, Braak LB and McKeith stage scores as wells as age, gender and MMSE score information. [file 40478_2020_989_MOESM9_ESM.docx]

Supplementary Table 1

| **Sample** | **Path Diag** | **NFT Braak** | **CERAD** | **NIA-RI** | **Thal Abeta** | **NIA-AA** | **Braak LB** | **McKeith** | **Age at death** | **Gender** | **Last MMSE** |
| --- | --- | --- | --- | --- | --- | --- | --- | --- | --- | --- | --- |
| 1 | nothing abnormal beyond age | 0 | neg | no probability of AD | 1 | not | 2 | no LBD | 70 | male | 30 |
| 2 | nothing abnormal beyond age | 2 | neg | no probability of AD | 2 | low | 0 | no LBD | 85 | female | 30 |
| 3 | nothing abnormal beyond age | 2 | neg | no probability of AD | 3 | low | 0 | no LBD | 73 | male | n/a but cognitively normal |
|  |  |  |  |  |  |  |  |  |  |  |  |
| 1 | AD | 6 | C | high probability of AD | 5 | high | 0 | no LBD | 80 | female | 18 |
| 2 | AD | 5 | C | high probability of AD | 5 | high | 0 | no LBD | 81 | female | 22 |
| 3 | AD | 6 | C | high probability of AD | 5 | high | 0 | no LBD | 84 | female | 0 |
| 4 | AD | 6 | C | high probability of AD | 5 | high | 0 | no LBD | 85 | male | 6 |
| 5 | AD | 6 | C | high probability of AD | 5 | high | 0 | no LBD | 88 | male | 20 |
